# Engineering light-inducible nuclear localization signals for precise spatiotemporal control of protein dynamics in living cells

**DOI:** 10.1038/ncomms5404

**Published:** 2014-07-14

**Authors:** Dominik Niopek, Dirk Benzinger, Julia Roensch, Thomas Draebing, Pierre Wehler, Roland Eils, Barbara Di Ventura

**Affiliations:** 1 German Cancer Research Center (DKFZ), Department of Theoretical Bioinformatics, Im Neuenheimer Feld 580, Heidelberg 69120, Germany; 2 Department of Bioinformatics and Functional Genomics, Synthetic Biology Group, Institute for Pharmacy and Molecular Biotechnology (IPMB) and BioQuant, University of Heidelberg, Im Neuenheimer Feld 267, Heidelberg 69120, Germany; 3 Present address: Department for Biosystems Science and Engineering, ETH Zurich, Mattenstrasse 26, 4058 Basel, Switzerland; 4 Present address: Department of Internal Medicine III, University Clinic Heidelberg, Im Neuenheimer Feld 669, 69120 Heidelberg, Germany

## Abstract

The function of many eukaryotic proteins is regulated by highly dynamic changes in their nucleocytoplasmic distribution. The ability to precisely and reversibly control nuclear translocation would, therefore, allow dissecting and engineering cellular networks. Here we develop a genetically encoded, light-inducible nuclear localization signal (LINuS) based on the LOV2 domain of *Avena sativa* phototropin 1. LINuS is a small, versatile tag, customizable for different proteins and cell types. LINuS-mediated nuclear import is fast and reversible, and can be tuned at different levels, for instance, by introducing mutations that alter *As*LOV2 domain photo-caging properties or by selecting nuclear localization signals (NLSs) of various strengths. We demonstrate the utility of LINuS in mammalian cells by controlling gene expression and entry into mitosis with blue light.

## Abstract (web summary)

Designing inducible and reversible nuclear localization signals would enable researchers to dissect and engineer cellular networks. Here Niopek *et al.* create a light-inducible nuclear localization signal to regulate gene expression and mitosis in mammalian cells, using blue light.

## Graphical abstract

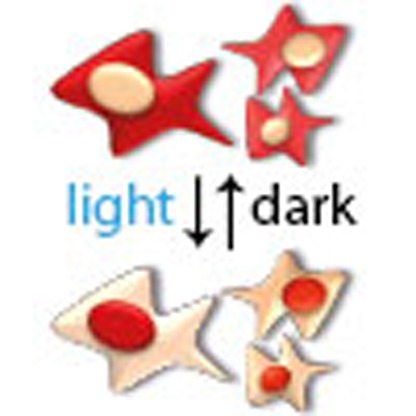

In recent years, the importance of nuclear translocation dynamics in the regulation of cellular pathways has been shown for key transcription factors (TFs) such as p53 (ref. 1) and nuclear factor-κB^2^,^3^,^4^, suggesting that tools to control such dynamics are widely needed in cell and developmental biology. Optogenetic approaches are particularly suited when high spatiotemporal control is required while having further advantages such as non-invasiveness, reversibility and fast response^5^. Two recent studies made use of light-inducible protein–protein interaction systems to accumulate a fluorescent protein in the nucleus^6^,^7^. While showing a strong nuclear accumulation of green fluorescent protein, the method based on UVR8 and COP1 is rather slow and irreversible, rendering it unsuitable for engineering complex import patterns. The second method, based on phytochrome B and PIF6, is fast and can be made reversible using 750 nm light. This requires a sophisticated microscopy setup when aiming at precise spatial control, an externally supplied chromophore and tagging the protein of interest with a large photosensory domain. Importantly, both systems are based on two individual components that need to be expressed at the right level to ensure precise and predictable control of nuclear translocation, which may be difficult to achieve.

Here we report the development of LINuS, a light-inducible, fully reversible and genetically encoded nuclear localization signal (NLS) based on a single, small tag, which can be fused to proteins of different sizes, origin and function, and which is independent of externally supplied chromophores. We show that LINuS can regulate the nuclear import of several functional proteins with light, working both in yeast and mammalian cells. To prove the usefulness of LINuS for cell biological applications, we created a system to trigger entry into mitosis of illuminated cells and engineered a TF that activates reporter gene expression after blue light illumination.

## Results

### Engineering LINuS based on monopartite NLSs

**Figure 1:**
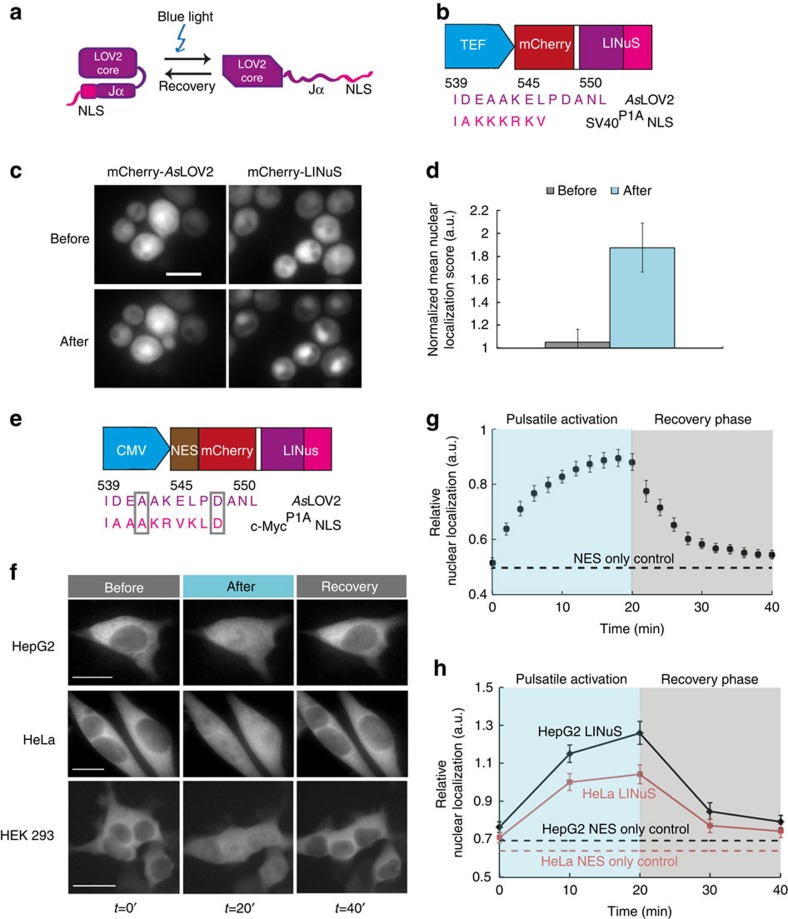
Development of light-inducible nuclear localization signals (LINuSs) for use in yeast and mammalian cells. (**a**) Schematic of LINuS function. In the dark state, the hybrid Jα helix is folded and interacts with the *As*LOV2 core domain. Upon blue light exposure, it unfolds rendering the NLS accessible to endogenous importins. (**b**) Schematic of the mCherry-LINuS expression construct based on the SV40 NLS for use in *S. cerevisiae*. TEF, constitutive promoter. White space, glycine–serine linker. LINuS, *As*LOV2 domain-NLS chimera (colour code as in **a**). Numbers show the position of the corresponding residues in the full-length *As*LOV2 domain. (**c**) Representative fluorescence microscopy images of *S. cerevisiae* cells expressing mCherry fused to the wild-type *As*LOV2 domain (truncated at residue 540) or to the LINuS tag, before and after continuous illumination with blue light for 10 min. Scale bar, 5 μm. (**d**) Quantification of the mean nuclear localization score for the mCherry-LINuS construct in **c**, normalized to the mean nuclear localization score for the mCherry-*As*LOV2 construct in **c**. Mean nuclear localization scores were calculated for a population (*n*≥36) of cells before and after blue light illumination. Error bars, s.d. of three independent experiments. (**e**) Schematic of the expression construct for mCherry-LINuS based on the mutated c-Myc^P1A^ NLS for use in mammalian cells. CMV, strong constitutive promoter. White spaces, glycine-serine linkers. NES, PKIt nuclear export sequence. Grey boxes highlight residues identical to those in the *As*LOV2 domain. (**f**) Representative fluorescence microscopy images of the indicated cell lines transiently transfected with the mCherry-LINuS construct shown in **e**. Illumination was performed with 1 s blue light pulses every 30 s, for 20 min followed by a 20-min recovery phase in the dark. Scale bars, 15 μm. Quantification of the import and export dynamics in HEK 293T cells (**g**) and HepG2 and HeLa cells (**h**). NES only control, construct shown in **e** without the NLS. As this construct remains cytoplasmic (Supplementary Fig. 6), only the initial value is shown for simplicity. Data represent mean±s.e.m. (*n*=21, two independent experiments (**g**) and *n*=20 cells, two independent experiments (**h**)).

To create a a light-inducible nuclear localization signal, we took advantage of the possibility to photo-cage small peptides using the LOV2 domain of *Avena sativa* phototropin 1 (*As*LOV2) (refs 8, 9). When introduced into the sequence of the C-terminal Jα helix of the *As*LOV2 domain, the NLS is concealed from the nuclear import machinery in the dark state (Fig. 1a). Upon blue light (~450–495 nm) absorption, the Jα helix unfolds and undocks from the *As*LOV2 core domain, allowing the NLS to be recognized and bound by the corresponding endogenous importins (Fig. 1a). The so-engineered *As*LOV2-NLS (termed as LINuS) can be used as a small tag to regulate the nuclear import of an arbitrary fused protein via light. We first investigated LINuS function in the yeast *Saccharomyces cerevisiae*. We selected the well-characterized NLS of the large T-antigen SV40 and explored different constructs where either the position of the peptide within the Jα helix or the first residue of the NLS itself was changed (Supplementary Fig. 1a). We fused each LINuS variant to the monomeric red fluorescent protein mCherry and expressed them in yeast from a constitutive promoter (Fig. 1b and Supplementary Fig. 1a).

Their small size is permissive for passive diffusion through the nuclear pore complex^10^, and the fusion proteins are slightly enriched in the nucleus even before blue light illumination (Fig. 1c and Supplementary Fig. 1b). This localization is not due to extensive exposure of the NLS in the dark state, as quantification of the nuclear localization (see Methods section) of each construct showed nuclear intensities for the dark state comparable to those of a mCherry-*As*LOV2 control bearing no NLS (Supplementary Fig. 1c). After illumination for 10 min, a clear nuclear accumulation of the fluorescence signal was observed for all constructs (Fig. 1c,d and Supplementary Fig. 1b,c). We selected the best performing construct (SV40 NLS var4) for further characterizations. To test reversibility, we repeatedly caused the nuclear accumulation of the mCherry-LINuS fusion protein by alternating between activation and recovery phases. We were thus able to accumulate mCherry-LINuS in the nucleus to the same extent at least for three consecutive cycles (Supplementary Fig. 1d,e).

### Characterization of LINuS in mammalian cells

We then decided to test LINuS in mammalian cells. To our surprise, constructs based on the SV40 NLS showed strong nuclear localization in the dark state (Supplementary Fig. 2a,b), suggesting that this particular NLS is too strong and its affinity for the import machinery wins over the caging ability of the Jα helix in HEK 293T (human embryonic kidney 293T) cells. Therefore, we tested different NLSs (Supplementary Fig. 2c) and found that a LINuS variant bearing a mutant c-Myc NLS (c-Myc^P1A^) could be accumulated in the nucleus after blue light illumination (Supplementary Fig. 2d).

Notably, tagging mCherry N-terminally is also possible (Supplementary Fig. 3a–c). This can be advantageous when working with proteins that do not tolerate C-terminal tags.

We further sought to decrease the non-negligible nuclear levels of the mCherry-LINuS construct in the dark state by introducing in the *As*LOV2 domain mutations that are known to increase docking or stability of the Jα helix^8^,^11^. These mutations decreased the background nuclear localization of the c-Myc^P1A^ NLS-based construct to the levels obtained for a no-NLS mCherry control (Supplementary Fig. 4b,c). Since they also decreased the response of the system to light, they should be preferred for applications when tightness is more critical than activation range. Mutations were not sufficient to render the SV40 NLS-based construct satisfactorily functional (Supplementary Fig. 4a,c).

To further decrease the background nuclear localization of mCherry-LINuS by passive diffusion and to allow the nuclear export of larger fusion proteins, we added a constitutive nuclear export signal (NES) to the construct (Fig. 1e and Supplementary Fig. 5a). As the steady state nucleocytoplasmic distribution of a given protein is dictated by its nuclear import and export rates, which are determined by the NLS and NES strength^12^, the right balance between these two sequences needs to be found. We selected two NESs of different strengths—the strong human immunodeficiency virus type 1 Rev protein (HIV) and the weaker truncated (cAMP-dependent protein kinase inhibitor alpha) PKI (PKIt) NESs—and coupled them to either wild type or strongly caged c-Myc^P1A^-based LINuS constructs (Supplementary Fig. 5a). These LINuS variants were all cytoplasmic in the dark state, yet the only one that could be significantly accumulated in the nucleus after activation was the construct bearing the PKIt NES with a LINuS based on the wild-type *As*LOV2 background (Supplementary Fig. 5b,d). A control mCherry fusion protein bearing only the PKIt NES and wild-type *As*LOV2 without any NLS did not accumulate in the nucleus after blue light illumination (Supplementary Fig. 6). Placing the NES at the C terminus of the LINuS tag is also possible, but leads to a lower activation range (Supplementary Fig. 5c,d). This result suggests that the location of the NES within the protein can also be used to modulate the tag behaviour.

Given its low nuclear localization background and its responsiveness to light, we selected the PKIt, c-Myc^P1A^-based construct for further characterization in several mammalian cell types (Fig. 1f). By using a pulsatile light regime, we found that mCherry-LINuS nuclear accumulation saturated quickly (~14 min; Fig. 1g,h). Notably, with this construct, we mostly reach an equilibration between cytosolic and nuclear localization during light activation (as indicated by values around 1.0 of the relative nuclear localization; Fig. 1g,h).

**Figure 2:**
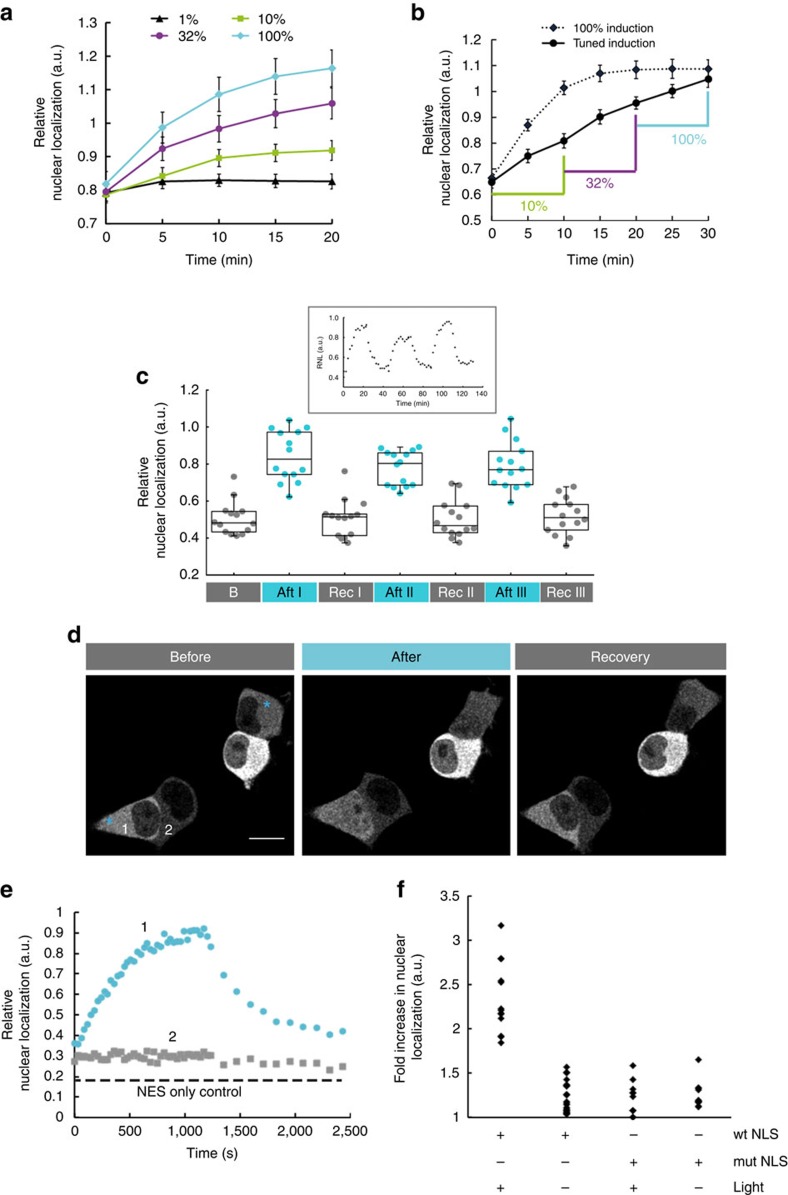
Tunability and reversibility of LINuS in mammalian cells. (**a**) Graph showing the relative nuclear localization of mCherry-LINuS over time for activation performed using increasing light intensities (1, 10, 32 and 100%). Transiently transfected HEK 293T cells were illuminated with 1 s blue light pulses of the indicated intensities every 30 s for 20 min. Data represent mean±s.e.m. (*n*=26 cells, two independent experiments). (**b**) As in **a**, but illumination was performed with 1 s blue light pulses every 30 s using either 100% light for 30 min (dashed line) or a sequence of pulses of the indicated increasing intensities (continuous line). Data represent mean±s.e.m. (*n*=20 cells, two independent experiments). (**c**) Box plot of the relative nuclear localization of mCherry-LINuS calculated for a population of HEK 293T cells over three cycles of 20 min illumination and 20 min recovery in the dark. Illumination was performed with 1 s blue light every 30 s for 20 min. Error bars indicate s.d. (*n*=13 cells, two independent experiments). Inset, relative nuclear localization of mCherry-LINuS over time for a representative cell. (**d**) Representative fluorescence time-lapse images of HEK 293T cells transfected with mCherry-LINuS and selectively illuminated to trigger nuclear import. Indicated cells (cyan asterisks) were illuminated with a blue (458 nm multiline argon) laser beam directed to a confined area in the cytoplasm. Light induction was performed by scanning the ROI for ~30 ms every 30 s for 20 min followed by 20 min dark recovery. Scale bar, 15 μm. (**e**) Quantification of the relative nuclear localization for the two indicated cells in **d**. Cell 2 was not illuminated. (**f**) Fold increase in nuclear localization of mCherry-LINuS in several individual cells activated for 20 min or non-activated. A LINuS construct bearing a mutated NLS (mut NLS, c-Myc^P1AK4A^) impaired in importin binding was used as control. Illumination was performed as described in **d**. Data from at least two independent experiments are shown. (**a**–**f**) mCherry-LINuS is the construct shown in Fig. 1e. wt, wild type.

Importantly, the extent of nuclear accumulation of LINuS may be varied by using different light intensities (Fig. 2a), which also enables the control of nuclear import dynamics (Fig. 2b). Together with the reversibility of LINuS activity (Fig. 2c), this leads to the possibility of precise temporal control of nuclear translocation. This holds true for the yeast mCherry-LINuS construct as well (Supplementary Figs 1d,e and 7a,b).

One important advantage of using light as trigger is the ability to accurately confine activation in space. Indeed, by directing a 458-nm laser beam onto single cells, we could initiate nuclear entry of mCherry-LINuS only in these cells, leaving the neighbouring cells unaffected (Fig. 2d–f). The observed nuclear translocation was not a side effect of the laser light (Fig. 2f and Supplementary Fig. 8).

### Optical control of mitotic entry

**Figure 3:**
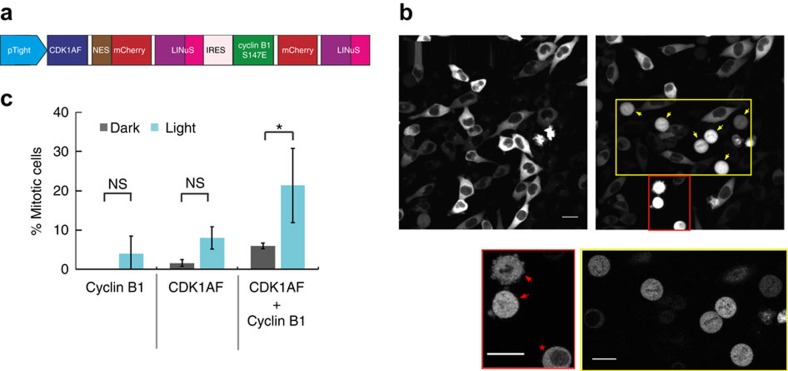
Light-induced entry into mitosis mediated by LINuS. (**a**) Schematic of the expression construct CDK1AF-mCherry-LINuS-IRES-Cyclin B1^S147E^-mCherry-LINuS. The white spaces represent glycine–serine linkers. (**b**) Top, representative average fluorescence intensity projections showing HeLa TetON cells transiently transfected with the construct in **a** before (left image) and after (right image) light activation. Yellow arrows depict cells that were counted as mitotic, based on their regular round shape, absence of a nucleus and, in some instances, presence of visible metaphase plates (shown in the zoomed image below, corresponding to one z-plane of the area in the yellow box above). Red box highlights cells that were not counted as mitotic despite being round, due to either the presence of a nucleus (red star, zoomed image) or blebs (red arrow, zoomed image). Light induction was performed by scanning the field of view for 8 s with 458 nm laser light every 30 s for 80 min. Scale bars, 20 μm. (**c**) Bar plot representing the percentage of mitotic cells in the dark and light for the indicated constructs. Error bars indicate s.d. (data from at least two independent experiments were pooled; *n*≥126 cells). *P<0.05 (Students *t*-test).

Even if the extent of nuclear accumulation after blue light illumination was modest, we thought that LINuS could be used to activate endogenous processes that require a timely nuclear translocation of a protein or a set of proteins whereby the activation threshold is relatively low. Cell division represents a very good example of one such process, with relatively low amounts of the cyclin B1-CDK1 complex accumulating in the nucleus determining the commitment of the cell to mitosis^13^, and the activation of early mitotic events such as nuclear envelope breakdown^14^. Therefore, we fused cyclin B1 and CDK1 to LINuS (Supplementary Fig. 9). We used a mutant CDK1 (CKD1AF), which is insensitive to its inactivating kinases Wee1 and Myt1 (ref. 15), thereby allowing it to be active throughout the whole cell cycle. While we could trigger nuclear import of CDK1AF-mCherry-LINuS fusion after blue light illumination (Supplementary Fig. 9), the cyclin B1-mCherry-LINuS construct only translocated into the nucleus upon mutation of the cyclin B1 cytoplasmic retention sequence (cyclin B1 S147E mutant^16^; Supplementary Fig. 9). We next examined whether we could cause illuminated cells to enter mitosis, regardless of the cell cycle phase they were in. To this aim, we transiently transfected HeLa TetON cells with a bicistronic construct expressing CDK1AF-mCherry-LINuS and cyclin B1^S147E^-mCherry-LINuS from the same pTight promoter (Fig. 3a), and induced their expression with doxycycline for 4–5 h (Supplementary Fig. 10). We then used a 458-nm laser to release caging of the NLS in both constructs and followed the cells by fluorescence time-lapse microscopy (Fig. 3b). Cells expressing only cyclin B1^S147E^-mCherry-LINuS or CDK1AF-YFP-LINuS served as control. We found that, while in the dark the number of mitotic cells resembled that of the controls, after blue light illumination ~21% of the cells entered mitosis (Fig. 3c). Notably, due to the inherent variability of protein levels among individual cells, not all illuminated cells go into mitosis (Fig. 3b).

### LINuS optimization using bipartite NLSs

As mentioned above, the current version of LINuS can be successfully applied to regulate processes for which the activation threshold is relatively low. Yet, there are applications for which a greater activation range is essential, thus we wanted to optimize LINuS to be able to accumulate more protein of interest into the nucleus.

**Figure 4:**
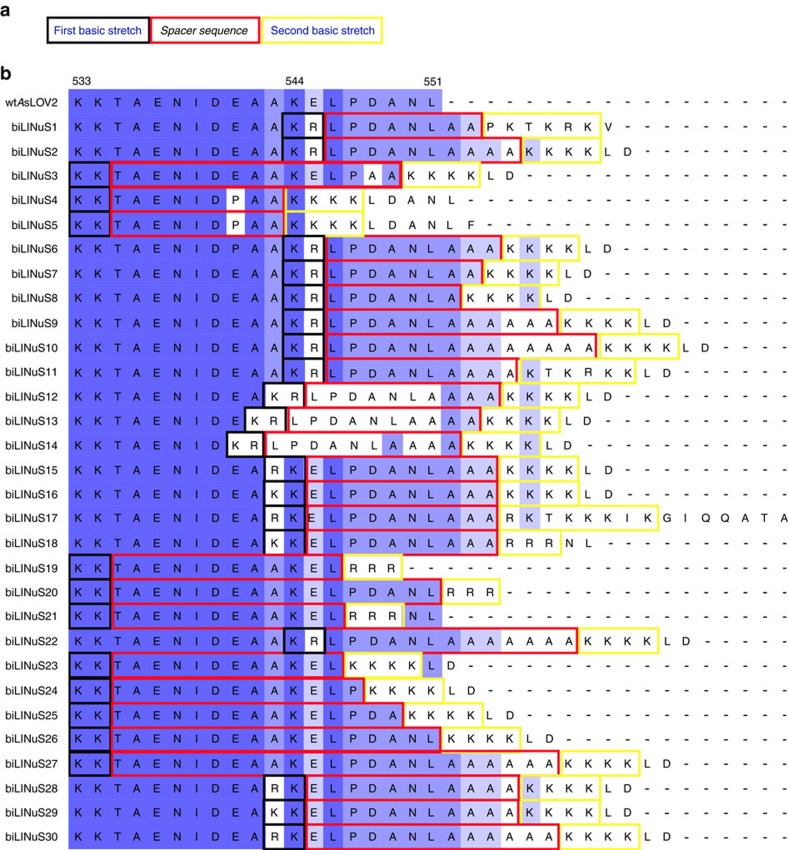
LINuS design based on bipartite NLSs. (**a**) Schematic representation of a bipartite NLS. (**b**) Multiple sequence alignment of the wild type *As*LOV2 C-terminal helix and corresponding biLINuS variants used in this study. Blue colouring of amino-acid residues indicates the degree of identity among the sequences shown (darker blue indicates higher identity). Colour code of the boxes as in **a**.

**Figure 5:**
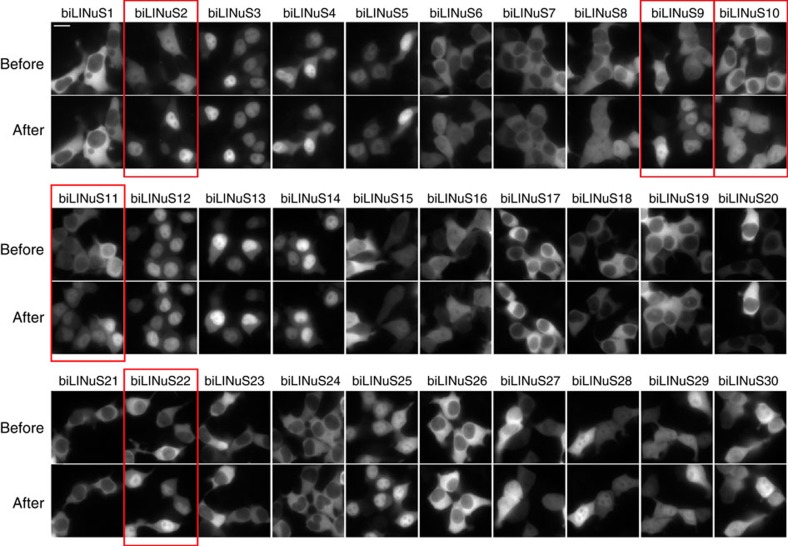
Qualitative assessment of biLINuS variants. Representative fluorescence microscopy images of HEK 293T cells transiently transfected with the indicated mCherry-biLINuS variants, before and after 15 min of illumination. All biLINuS variants carry a constitutive PKIt NES. Illumination was performed with 1 s blue light pulses every 30 s. Red boxes indicate mCherry-biLINuS variants quantified in Fig. 6a. Scale bar, 15 μm.

**Figure 6:**
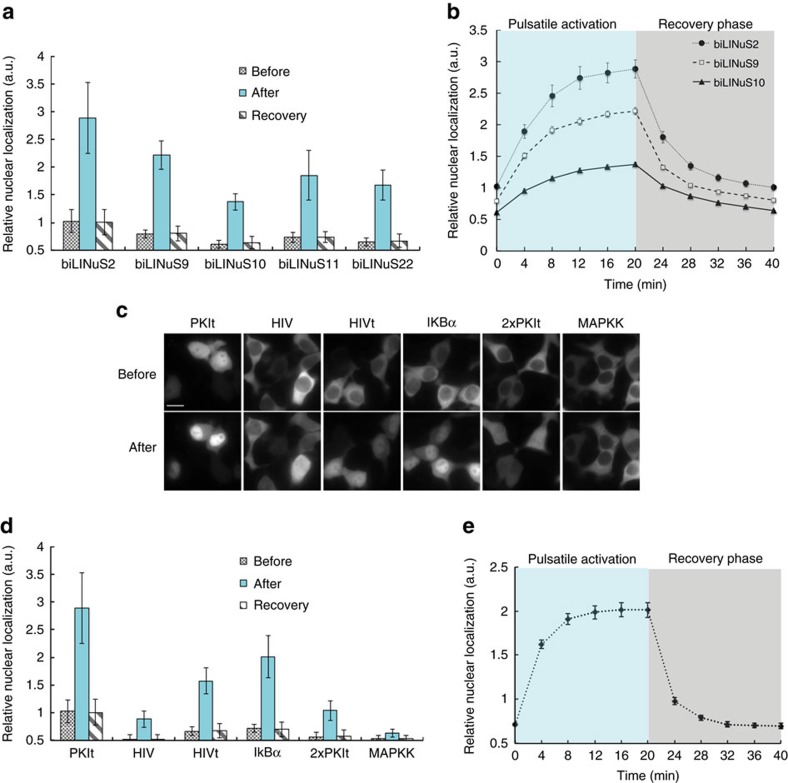
Characterization and optimization of biLINuS variants. (**a**) Quantification of the relative nuclear localization of the indicated mCherry-biLINuS variants in HEK 293T cells before activation (Before), after 20 min of illumination (After) and after recovery phase in the dark (Recovery). Data represent the mean ± s.d. (*n*=20 cells, two independent experiments). (**b**) Quantification of import and export dynamics of the indicated mCherry-biLINuS variants in HEK 293T cells. Data represent the mean± s.e.m. (*n*=20 cells, two independent experiments). (**a**,**b**) All biLINuS variants carry a constitutive PKIt NES. (**c**) Representative fluorescence microscopy images of HEK 293T cells transiently transfected with mCherry-biLINuS2 variant bearing the indicated NES before and after 20 min of illumination. 2xPKIt indicates two repeats of the PKIt NES. Scale bar, 15 μm. (**d**) Quantification of the relative nuclear localization of biLINuS2 variants with the indicated NESs in HEK 293T cells before activation (Before), after 20 min of illumination (After) and after recovery phase in the dark (Recovery). Data represent the mean±s.d. (*n*=20 cells, two independent experiments). (**e**) Quantification of import and export dynamics of the mCherry-biNLS2 variant carrying the IkBα NES in HEK 293T cells. Data represent the mean±s.e.m. (*n*=20 cells, two independent experiments). (**a**–**e**) Illumination was performed with 1 s blue light pulses every 30 s for 20 min, followed by 20 min recovery phase in the dark.

The success of the approach used in this study strictly relies on the ability to incorporate the NLS sequence into the Jα helix disrupting this latter as little as possible. We therefore thought that using bipartite NLSs could allow for stronger caging of the NLS in the dark all the while resulting in higher activation by light. Indeed, bipartite NLSs are composed of two stretches of basic residues, separated by a linker region of variable composition and length^17^ (Fig. 4a). This means that we can mutate fewer residues to incorporate the first basic stretch into the Jα sequence, leaving the second basic stretch outside of the helix. We generated a small library of 30 bipartite LINuS (biLINuS) variants around the bipartite NLSs of nucleoplasmin^18^,^19^,^20^ and human interleukin-5 (ref. 21). These constructs contain bipartite NLS-like sequences that differ in either amino-acid composition of first and second basic stretch, spacer sequence or position of these features within the Jα helix (Fig. 4b). We transiently transfected each variant into HEK 293T cells and observed qualitatively the extent of activation after blue light illumination (Fig. 5 and Table 1). Strikingly, we found five variants (biLINuS2, biLINuS9, biLINuS10, biLINuS11 and biLINuS22) that led to nuclear accumulation of the mCherry target protein beyond that achieved with the c-Myc^P1A^-based LINuS construct (compare Fig. 1f with Fig. 5). The quantification of the relative nuclear localization before and after blue light induction for these five variants show that the activation range is higher than that obtained with the c-Myc^P1A^-based LINuS construct (compare Fig. 6a with Fig. 1g,h). All constructs fully recovered their initial localization after a recovery phase in the dark (Fig. 6a). Confirming the bipartite nature of the NLS used in these biLINuS constructs, the light-dependent import of mutants for which positive residues in either the first or second basic stretch are exchanged with alanines is strongly impaired (Supplementary Fig. 11a,b). We further quantified the kinetics of nuclear import/export for three variants (Fig. 6b) and found half times of nuclear import and export of about 4 min for all constructs. Therefore, the major difference among these constructs is the initial nuclear localization level and the extent of nuclear accumulation achieved after light activation (Fig. 6a).

### LINuS optimization using NESs of different strength

The biLINuS2 variant leads to an almost complete nuclear accumulation of mCherry, its drawback being the elevated nuclear levels in the dark state (Figs 5 and 6a,b). Despite being suitable for applications in which the protein of interest is nuclear before the signalling event and accumulates in the nucleus after receiving a stimulus^22^,^23^, we wondered whether it would be possible to modify this construct in order to have it mostly cytoplasmic in the dark without significantly affecting the activation range. To this aim, we exchanged the PKIt NES for several different NESs, some of which were very strong and others weaker (Fig. 6c). Interestingly, two of the tested NESs gave rise to a modified biLINuS2 tag that started from cytoplasmic distribution, but could still be strongly accumulated in the nucleus with light (Fig. 6d). We further quantified the import/export kinetics of one of these variants and found them to be the fastest among all LINuS constructs generated (Fig. 6e).

Taken together, these results indicate that it is possible to tinker with LINuS in various ways (for example, by choosing the NLS, by locating it at different positions into the Jα helix and by choosing the NES) to obtain the most suitable tag for a specific application.

### Light-induced reporter gene expression

**Figure 7:**
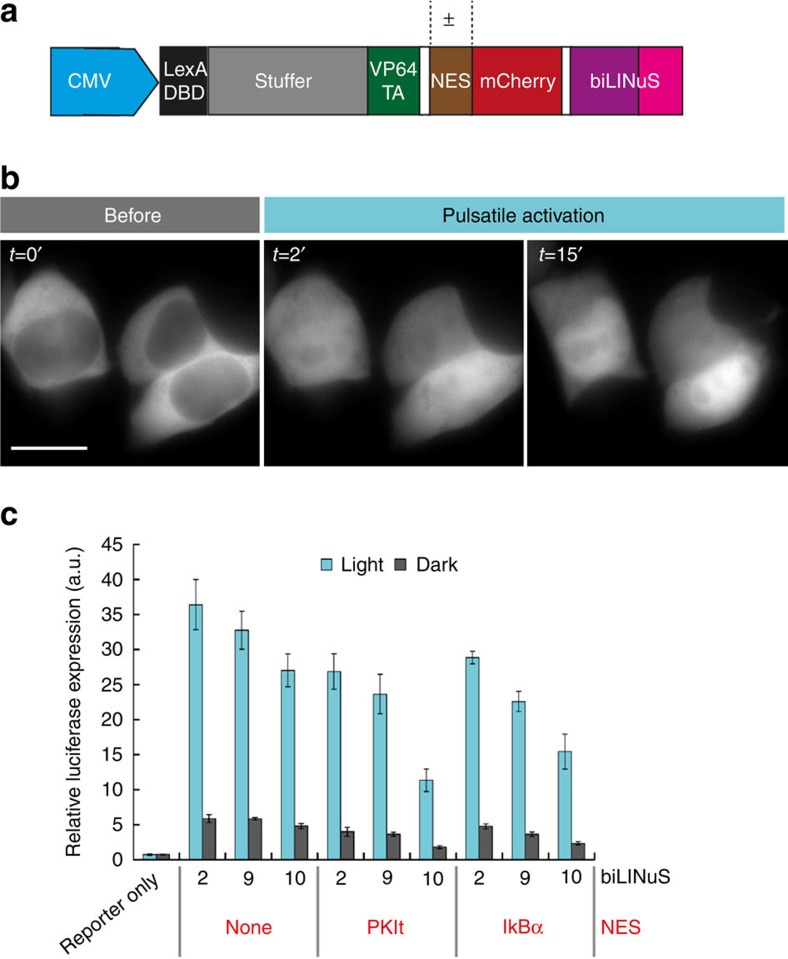
Light-induced gene expression mediated by biLINuS. (**a**) Schematic of the expression construct for biLINuS-based synthetic mammalian TFs. Besides bearing different biLINuS variants, they differ by the presence or absence of an NES and by the nature of the NES, when present. DBD, DNA-binding domain. TA, transactivation domain. LINuS indicates the *As*LOV2 domain-NLS chimera (colour code as in Fig. 1a). The white spaces represent glycine–serine linkers. (**b**) Representative fluorescence microscopy images of HEK 293T cells transiently transfected with biLINuS10 variant of the construct in (**a**) lacking an NES (103 kDa) before and after illumination with blue light. Illumination was performed with 1 s blue light pulses every 30 s, for 15 min. Scale bar, 15 μm. (**c**) Quantification of luciferase activity in HEK 293T cells transiently co-transfected with the indicated TF, a reporter construct consisting of the firefly luciferase gene driven from a minimal promoter coupled to four LexA-binding sites, and a constitutive *renilla* expression construct. Illumination started 15 h post transfection and was performed for 24 h with constant blue light (*λ*_max_ =460 nm, ~10 μmol m^−2^ s light intensity). Firefly luciferase activity was normalized to *renilla* luciferase in each sample. Data represent mean±s.d. (three independent experiments).

As gene expression is a process that requires the presence in the nucleus of the corresponding TF, we thought it was a natural application for LINuS. As a proof of principle, we engineered a synthetic TF comprising a DNA-binding domain of bacterial origin^24^, a stuffer protein to increase the TF size, the VP64 transactivation domain and LINuS, without or with constitutive NESs of different strength (Fig. 7a). We designed these constructs following the principle of modularity to allow future users to easily substitute a protein of interest for the TF and try out several combinations of NLS/NES (Supplementary Fig. 12a; Supplementary Methods). The TF (~103 kDa) translocated into the nucleus upon light induction (Fig. 7b). We detected a light-dependent activation of gene expression for all constructs tested (Fig. 7c and Supplementary Fig. 12b), with a maximal sevenfold increase. The background and the extent of activation by light depend on the NLS and NES selected (Fig. 7c and Supplementary Fig. 12b), hence the right TF-LINuS construct should be selected depending on the specific application. Control constructs in which the NLS is impaired by mutation show impaired light-induced transcription (Supplementary Fig. 12c), while removing the NLS altogether abolishes any light effect (Supplementary Fig. 12d). The light regime used in this experiment did not cause any detectable toxicity to the cells (Supplementary Fig. 12e).

## Discussion

Here we reported the development and showcased the applicability of LINuS, a tool for regulating nuclear import of proteins with blue light. We have shown LINuS function in yeast and mammalian cells, but given that this method relies solely on the interaction between the photocaged NLS and the endogenous import machinery, it is likely to be functional also in other eukaryotic systems. The specific NLS/NES sequences might need to be adjusted to obtain optimal performance in each cell type, as exemplified by the difference in strength that we observed for the SV40 NLS in yeast and mammalian cells (compare Supplementary Fig. 1b with Supplementary Fig. 2b), or by the difference in activation range achieved by the same construct in different cell lines (Fig. 1f–h). This is likely due to the variability of the import/export machineries in different cell types, for example nature as well as absolute and relative abundance of import and export factors. Nevertheless, we showed here that the general design of an NLS photocaged within the Jα helix of the *As*LOV2 domain is possible in various cellular contexts, indicating that LINuS is a highly versatile optogenetic tool.

Even if in this work we only described nuclear accumulation of exogenously expressed fusion proteins, we speculate that LINuS can be used to regulate the import of genomically integrated fusion proteins, albeit the illumination regime or NLS/NES selection might need to be appropriately adjusted. However, a rigorous investigation of the applicability of LINuS for regulation of endogenous proteins needs to be performed.

While optogenetic protein–protein interaction systems could be adapted to obtain nuclear import systems triggered by light, they would bear several disadvantages over LINuS, such as higher number of components and larger size of protein tags. Adapting existing optogenetic systems to nuclear translocation would require substantial engineering of these methods, and their suitability for controlling nuclear translocation of functional proteins in cell biological applications would have to be demonstrated. LINuS is a minimal system for inducible nuclear localization of arbitrary proteins based on a single, small protein tag and is preferable over other systems, based on light-induced protein dimerization, given its compactness and simplicity.

Conceptually, LINuS can be used to regulate the import dynamics of any protein of interest, regardless of its size or function. Indeed, here we showed that we could regulate via light the function of a large TF, a regulatory protein and a kinase. Notably, contrary to all methods described so far, LINuS-mediated gene expression is not restricted to modular, synthetic TFs composed of separated DNA-binding and transactivation domains that need to come together to reconstitute a functional unit. LINuS can, thus, be used with intact TFs. For most proteins, it will be necessary to mutate endogenous NLSs and/or NESs to be able to control their nuclear entry only via light. Fortunately these sequences and corresponding required mutations are often known or can be predicted, making LINuS a strong candidate for externally triggering the nuclear import of any protein of interest.

In conclusion, here we have created a small library of photosensitive NLSs that can be fused to a protein of interest to regulate its nuclear import with blue light. The different variants offer a wide range of properties (for example, biLINuS2 high activation range, LINuS c-Myc^P1A^ or biLINuS10 low background) that can be further tuned by the user, effectively expanding the number of tags to chose from to match it to the specific application. The tool presented here will, thus, fundamentally increase our abilities to study the importance of protein dynamics in cell biology.

## Methods

### Plasmid construction

Plasmid construction was performed using standard restriction enzyme cloning. Details on the cloning procedures and a list of all constructs and oligonucleotides used or constructed in this study are shown in Supplementary Methods and Supplementary Tables 1 and 2 Constructs created in this study are available through the Helmholtz centres’ repository of biological parts, HeRBi (www.herbi.kit.edu).

### Characterization of nuclear translocation in yeast

For all experiments the *S. cerevisiae* strain SEY6210 was used. Transformations with LINuS expression plasmids were performed using the high efficiency, LiAc/SS carrier DNA/PEG method^25^. Transformed yeast strains were grown in synthetic dropout medium without histidine (SD-His). For microscopy analysis, overnight cultures were diluted to an OD_600_ of 0.2 with fresh SD-His medium, and were grown to an OD_600_ between 0.6 and 0.8. Cells were applied to a thin 1% agarose pad (SD-His). The cell suspension was allowed to dry for few minutes and a coverslip was placed on top of the agarose pad. Fluorescence microscopy pictures were acquired at room temperature using a DeltaVision microscopy system (Applied Precision) consisting of an Olympus IX inverted microscope (Olympus) equipped with a cooled CoolSnap HQ CCD camera (Photometric) and an HBO 100W mercury arc lamp light source (Olympus). Filter sets used have the following wavelengths/bandwidth (in nm): excitation 490/20, emission 528/38 for FITC and excitation 555/28 and emission 617/73 for mCherry. A × 63/1.40 numerical aperture (NA) oil objective (Olympus) was used for image acquisition. Cells were focused using the mCherry channel in order to avoid premature activation of LINuS due to white light exposure. For initial characterization, light induction of LINuS constructs was performed by constant illumination with 490 nm light (FITC channel) with 100% intensity for 10 min. Before and after illumination, images were acquired using the mCherry channel in order to analyse mCherry-LINuS localization. Resolve3D software was used for image acquisition. For time-lapse microscopy analysis of mCherry-LINuS translocation, light induction was performed by illumination with 490 nm light pulses (FITC channel). Each light pulse was followed by image acquisition in the mCherry channel. Both light induction and image acquisition were performed in a Z-stack of 15 sections with 0.4 μm step size in order to minimize uneven induction and avoid problems with the quantification of nuclear fluorescence due to defocusing during the period of the time lapse. The reported light-induction pulse lengths correspond to the sum of the exposure times for each section. Illumination with different light intensities was done using neutral density filters. For the analysis of the decay in nuclear fluorescence, images were only acquired using the mCherry channel. Image analysis was performed using ImageJ software^26^. Nuclear localization was analysed using a previously described localization score that is defined as the difference between the mean intensity of the five brightest percent of pixels in the cell and the mean intensity of the rest of the pixels in the cell, normalized by the mean pixel intensity of the cell^27^. Yeast cells were segmented manually. The TurboReg plugin^28^ was used to align images of a given time series in order to correct for stage drift. Regions of interest (ROIs) were adjusted manually for a given time series if necessary. The localization score was calculated for every individual cell/ROI at every experimental time point after subtraction of image background. For each experiment and condition, at least 30 cells were analysed.

### Nomenclature for light induction experiments

To describe experiments for which the same cells were imaged before and after light induction, we refer to ‘before’, ‘after’ (last time point during the light activation phase) and ‘recovery’ (last time point after a recovery phase in the dark). When more images/data points are shown, we use the terms ‘pulsatile activation’ and ‘recovery phase’. To indicate experiments where two separate groups of cells were either exposed to light or kept in the dark, we use the terms ‘light’ and ‘dark’.

### Mammalian cell culture and transfection

HEK 293T and HepG2 (human hepatocellular carcinoma) cell lines were kindly provided by Dirk Grimm (University of Heidelberg). HeLa TetON (human cervix carcinoma) cell line stably expressing the Tet repressor was kindly provided by Ingrid Hoffmann (German Cancer Research Center, Heidelberg). All cells were maintained at 37 °C and 5% CO_2_ in phenol red-free Dulbecco’s Modified Eagle Medium supplemented with 10% fetal calf serum (Biochrom AG, Berlin, Germany), 2 mM L-glutamine (Invitrogen/Gibco), 100 U ml^−1^ penicillin and 100 μg ml^−1^ streptomycin (Invitrogen/Gibco). The medium for the HeLa TetON cell line was additionally supplemented with 300 μg ml^−1^ G418 (Roth). For fluorescence microscopy analysis of mCherry-LINuS and mCherry-biLINuS variants, cells were seeded into 35 mm glass bottom Cellview cell-culture dishes (Greiner BIO-ONE) or Nunc 8-well Lab-Tek chamber slides (Thermo Scientific). The next day, transfection was performed using Lipofectamine 2000 (Invitrogen) or JetPrime (PolyPlus Transfection) according to the manufacturer’s instructions. For Lipofectamine 2000 transfection in HEK 293T, HepG2 and HeLa TetON, 2–3 μg of total DNA per 35 mm dish and 500 ng per Lab-Tek well were used. mCherry-LINuS and mCherry-biLINuS expression constructs were transfected alongside with pBlueScriptIIS/K stuffer plasmid in a ratio of 1:10–1:20, respectively. For shuttling experiments in HepG2, the ratio was 1:1. For JetPrime transfection in HEK 293T, 500 ng of total DNA were used per 35 mm dish, and the ratio of mCherry-LINuS or mCherry-biLINuS to pBlueScriptIIS/K stuffer plasmid was 1:10. Microscopy analysis was performed ~24 h post transfection.

### Characterization of LINuSs in mammalian cell lines

Fluorescence microscopy images were acquired at 37 °C and 5% CO_2_ using the same microscopy setup used for yeast. For experiments shown in Fig. 1f (HepG2 and HeLa cells), Figs 1h and 2a,b, a × 40 (UApo, NA 1.35) air objective was used instead of the × 63/1.40 NA oil objective in order to increase the number of cells per field of view. Time-lapse analysis of LINuS-mediated translocation was performed as described for yeast with the following changes: if not indicated differently in the figure legends, light induction was performed with a 1-s blue light pulse of 100% intensity every 30 s using the FITC channel. Neutral density filters were used to reduce the light intensity from 100 to 32, 10 or 1% (Fig. 2a,b). For the analysis of the decay in nuclear fluorescence, mCherry images were taken every 2 min to reduce photobleaching. For the quantification of nuclear translocation, background-subtracted images were quantified by drawing a ROI in the cytoplasm and nucleus of each cell, and the ROIs mean mCherry fluorescence intensity was calculated using ImageJ. The reported relative nuclear localization is defined as the ratio between mean nuclear and cytoplasmic fluorescence intensities. In some cases, the fold increase in nuclear localization was calculated as the ratio between the relative nuclear localization before and after illumination. mCherry-LINuS translocation experiments in single cells were performed using the ROI Scan function of a Leica Sp5 confocal microscope equipped with a PL Apo CS × 40 oil objective (NA=1.3; Fig. 2d–f). A circular ROI with an area of ~32 μm^2^ positioned in the cytoplasm was scanned with 458 nm light of a multiline argon laser. The light intensity was set to 2–2.5 μW as measured with a Laser Power and Energy Meter (Nova). Cells were activated with 30 ms light pulses every 30 s for 20 min. During the recovery phase cells were imaged every 2 min with 561 nm laser light only. All experiments were carried out at 37 °C and 5% CO_2_.

### Induction of mitotic entry

HeLa TetON cells were seeded in 35 mm glass bottom dishes and co-transfected with 250 ng Cyclin B1^S147E^-mCherry-LINuS and 1,750 ng pBluescript stuffer, 250 ng CDK1AF-eYFP-LINuS and 1,750 ng pBluescript stuffer, and 1,000 ng CDK1AF-mCherry-LINuS-IRES-CyclinB1^S147E^ mCherry-LINuS and 1,000 ng pBluescript stuffer, respectively, using JetPrime transfection reagent. Expression was induced in unsynchronized cells by adding doxycycline (1 μg ml^−1^) 24 h post transfection. After 4–5 h of doxycycline induction, cells were imaged using a Leica SP5 confocal microscope as described above. LINuS translocation was achieved by scanning all cells in the field of view with 8 s 458 nm light pulses every 30 s for 80 min. Z-stack images were acquired before and after light activation. Cells were counted as mitotic if they had a regular round shape, showed no signs of blebbing and the absence of a nucleus. Non-activated control cells were imaged with 561 nm light only.

### Western blotting

Hela TetON cells were transfected with CDK1AF-mCherry-LINuS, CyclinB1^S147E^ mCherry-LINuS and CDK1AF-mCherry-LINuS-IRES-CyclinB1^S147E^ mCherry-LINuS. Cells were lysed on ice for 10 min in lysis buffer (20 mM Tris-HCl, pH 7.4, 1% Triton X-100, 10% glycerol, 150 mM NaCl, 1% phenylmethylsulfonyl fluoride (PMSF), 1 % Benzonase (Novagen) and 1 Complete Mini Protease Inhibitor tablet (Roche)). Proteins were fractionated by SDS-PAGE and transferred to a nitrocellulose membrane. mCherry fusion proteins were detected using an anti-mCherry antibody (Abcam, ab125096, diluted 1:666) and Beta actin was detected using an anti-Beta Actin antibody (Abcam, ab8226, diluted 1:5,000). As secondary antibody, a goat anti-mouse IgG (H+L)-HRPO (Dianova, 115-035-003) was used. Chemiluminescence signal was detected using an enhanced chemiluminescence system (Amersham Biosciences) and the ChemoCam Imager (Intas).

### Luciferase reporter assay

HEK 293T cells seeded in clear-bottom 96-well plates (~10,000 cells per well) were co-transfected with 20 ng of LINuS-based TF variants, 20 ng of LexA-dependent firefly luciferase reporter, 0.1 ng of a constitutive *renilla* expression construct (pRL-TK, Promega) and 40 ng of stuffer DNA (pBlueScriptIIS/K) using JetPrime (only used for experiments shown in Fig. 7c and Supplementary Fig. 12d) or Lipofectamine 2000. Fifteen hours post transfection, continuous illumination of plates with 460 nm blue light was performed for 24 h using a custom-made LED device composed of six high-power LEDs type CREE XP-E D5-15 (LED-TECH.DE; light intensity was 10 μmol s^−1^ m^2^ as measured with a LI-COR LI-250A Light Meter) or kept in the dark. Cells were harvested into lysis buffer supplied with the Dual-Glo luciferase assay kit (Promega), and firefly and *renilla* luciferase activities were quantified using a Mithras LB 940 plate reader with automated injectors.

### MTT cell proliferation and viability assay

HEK 293T cells seeded in clear-bottom 96-well plates (~10,000 cells per well) were co-transfected with 20 ng of LINuS-based TF variants, 20 ng of LexA-dependent firefly luciferase reporter, 0.1 ng of a constitutive *renilla* expression construct (pRL-TK, Promega) and 40 ng of stuffer DNA (pBlueScriptIIS/K) using Lipofectamine 2000. Fifteen hours post transfection, continuous illumination of plates with 460 nm blue light (10 μmol s^−1^ m^2^) was performed for 24 h. Subsequently, an MTT assay was performed using the MTT Cell Proliferation Assay Kit (Cayman Chemical Company) following the manufacturer’s protocol. Absorbance at 570 nm was measured using infinite M200 (TECAN) plate reader. Non-illuminated cells served as control. Non-illuminated cells treated with different concentrations of Triton X-100 (Sigma) directly before MTT addition served as control for general assay performance and sensitivity.

### Statistical analysis

Percentage of mitotic cells (Fig. 3c) is reported as mean over independent experimental replicates (independent transfections, sample preparations and measurements on different days). Uncertainties in these values are reported as the s.d. of the different experimental replicates. A two-tailed Student’s *t*-test was used in order to test the differences in the percentage of mitotic cells between samples under light and dark conditions for statistical significance (*P*<0.05).

## Author contributions

D.N., D.B., R.E. and B.D.V. conceived the study; D.N., D.B., T.D., J.R. and B.D.V. designed experiments; D.N., D.B., T.D., J.R. and P.W. performed experiments; D.N., D.B., T.D., J.R. and B.D.V. performed analyses; B.D.V. and R.E. wrote the manuscript with support from all authors.

## Additional information

**How to cite this article:** Niopek, D. *et al.* Engineering light-inducible nuclear localization signals for precise spatiotemporal control of protein dynamics in living cells. *Nat. Commun.* 5:4404 doi: 10.1038/ncomms5404 (2014).

## Supplementary Material

Supplementary Information Supplementary Figures 1-12, Supplementary Tables 1-2, Supplementary Methods and Supplementary References

## Figures and Tables

**Table 1:** Qualitative comparison of different bipartite LINuS variants.



