# Supplementary material for: Engineering light-inducible nuclear localization signals for precise spatiotemporal control of protein dynamics in living cells
Source: Nat Commun. 2014 Jul 14;5:4404. doi: 10.1038/ncomms5404 (PMC4104460; doi:10.1038/ncomms5404)
Supplement: Supplementary Information — Supplementary Figures 1-12, Supplementary Tables 1-2, Supplementary Methods and Supplementary References [file ncomms5404-s1.pdf]

Supplementary Figures

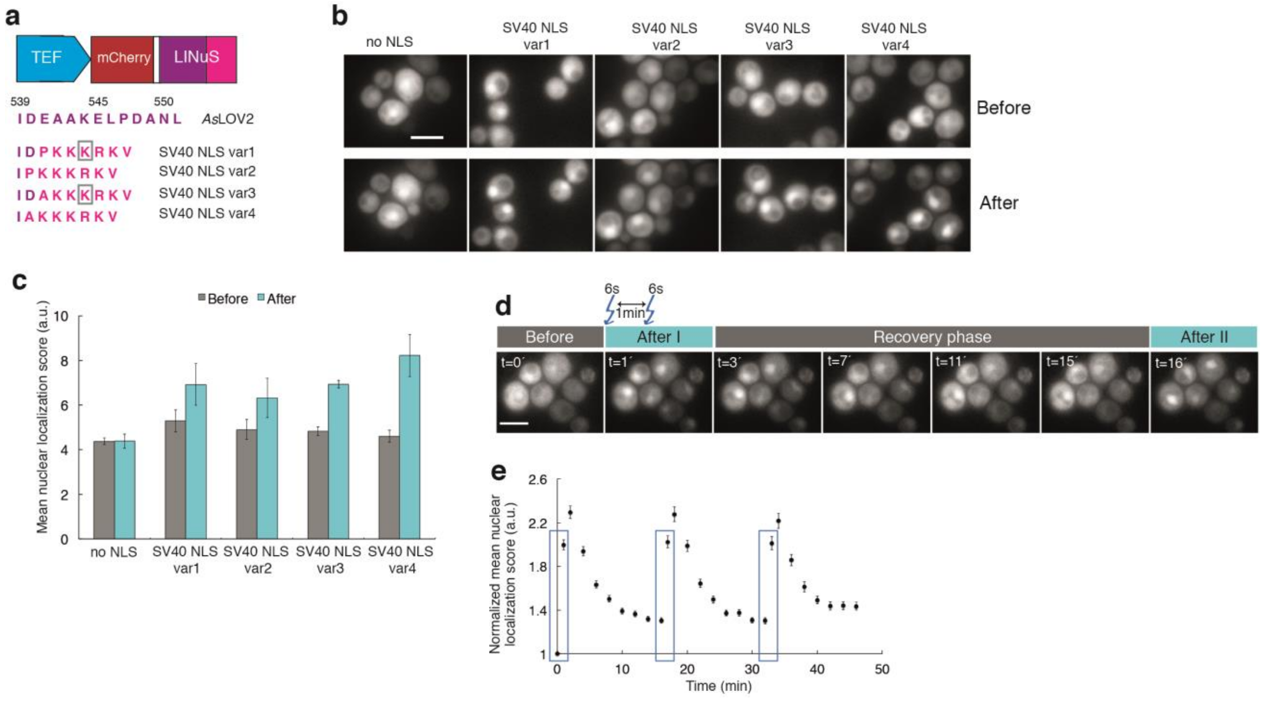

Supplementary Figure 1: Characterization of LINuS variants in *S. cerevisiae*. (**a**) Schematic of LINuS expression construct for use in *S. cerevisiae*. TEF is a strong constitutive promoter. The white space represents a glycine-serine linker. Variants 3 and 4 contain the SV40^{P1A} NLS. Numbers show the position of the corresponding residues in the full-length LOV2 domain. Grey boxes highlight residues that are identical to those in the *As*LOV2 domain. (**b**) Representative fluorescence microscopy images of the indicated constructs before and after constant blue light illumination for 10 min. no NLS, mCherry fused to *As*LOV2 truncated at residue 540. Scale bar, 5 µm. (**c**) Mean nuclear localization scores (Methods) were calculated for a population (n ≥ 36) of cells before and after blue light illumination. Error bars, s.d. of 3 independent experiments. (**d**) Representative time-lapse fluorescence microscopy images of mCherry-LINuS SV40 NLS var4 in yeast cells. Light activation was performed as indicated, followed by a recovery

dark phase and a second activation. Scale bar, 5 µm. (**e**) Quantification of the mean nuclear localization score for construct shown in (**d**), normalized by the score at time = 0, for a population of cells that was repeatedly activated (2 blue light pulses of 6 sec, with 1 min interval; blue boxes). Data represent mean ± s.e.m. (n=71 cells, two independent experiments).

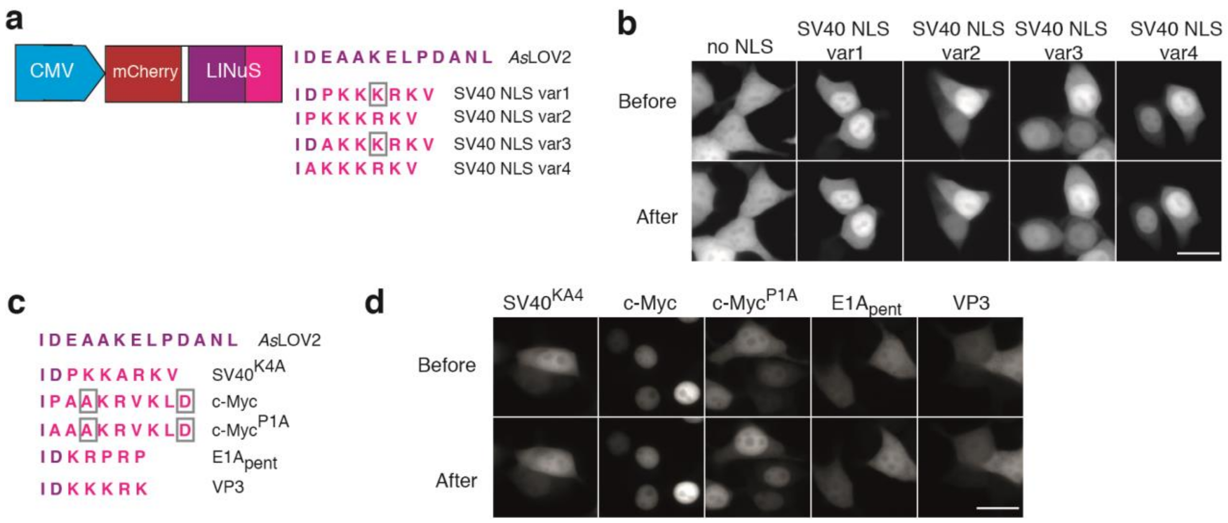

Supplementary Figure 2: Characterization of LINuS variants in HEK 293T cells. (**a**) Schematic of mammalian LINuS expression constructs. CMV, strong constitutive promoter. The white space represents a glycine-serine linker. (**b**) Representative fluorescence microscopy images of the indicated mCherry-LINuS constructs transiently transfected in HEK 293T cells. Illumination was performed with constant blue light for 20 min. (**c**) Different LINuS variants based on the indicated NLSs. (**d**) As in (**b**) but for the constructs in (**c**). Scale bars, 20 µm. (**a** and **c**) Grey boxes highlight residues that are identical to those in the *As*LOV2 domain

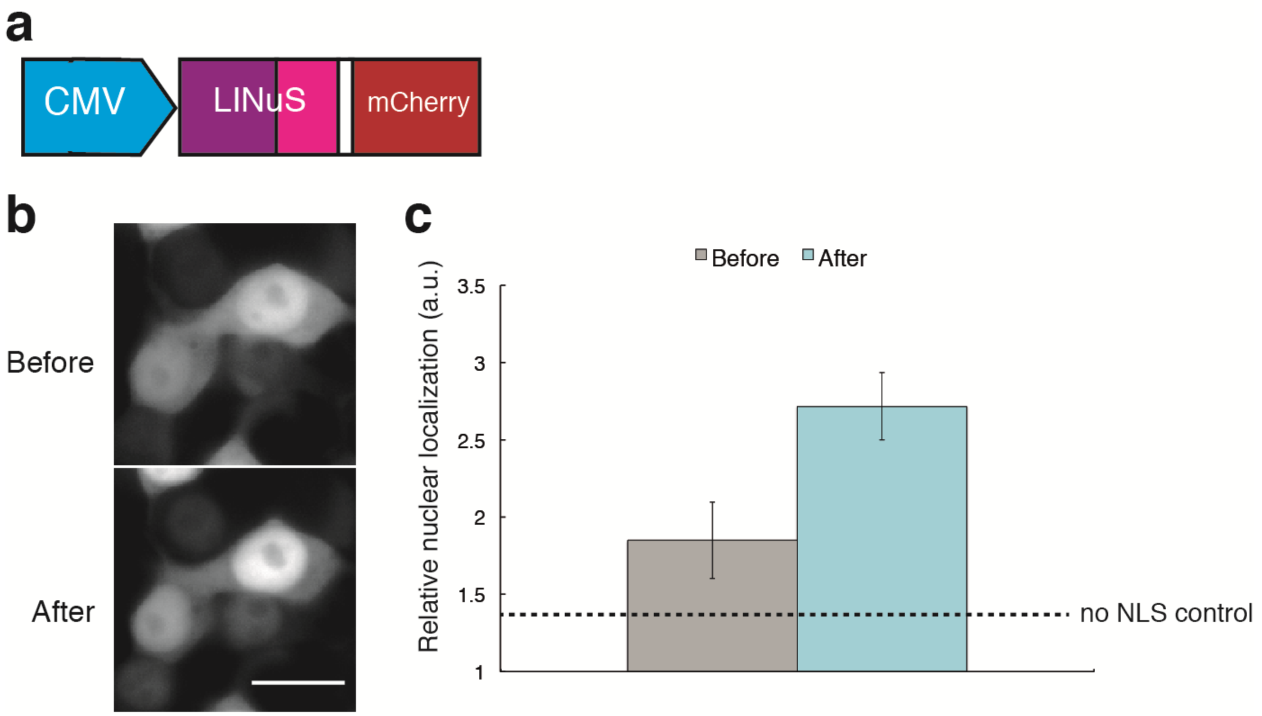

Supplementary Figure 3: N-terminal tagging with LINuS is possible. (a) Schematic of the N-terminal LINuS-mCherry fusion construct based on the c-Myc^{P1A} NLS. The white space represents a GS-linker. **(b)** Representative fluorescence microscopy images of the construct in (**a**) transiently transfected in HEK 293T cells before and after constant blue light illumination for 20 min. Scale bar, 20 µm. (**c**) Quantification of the relative nuclear localization obtained with the construct in (**a**). Data represent mean ± s.d. ($n \geq 6$ cells, 3 independent experiments). no NLS, mCherry fused to *As*LOV2 truncated at residue 540.

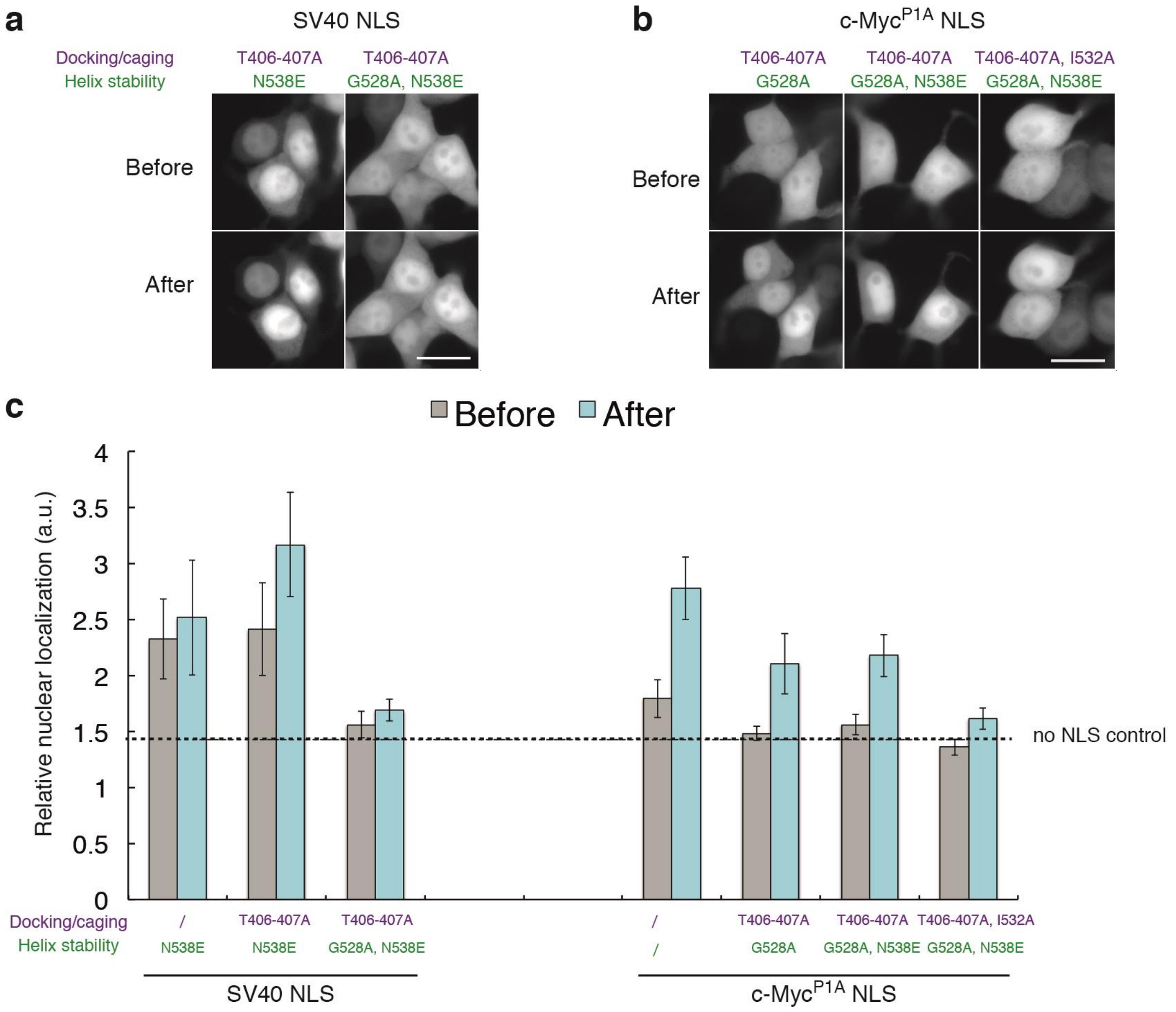

Supplementary Figure 4: Tunability of LINuS by *As*LOV2 mutations. (**a-b**) Representative fluorescence microscopy images of the indicated mCherry-LINuS constructs transiently transfected in HEK 293T cells. Illumination was performed with constant blue light for 20 min. Docking/caging strength increasing as well as helix stabilizing mutations are indicated[1]. Scale bar, 20 μm. (**a**) SV40 NLS indicates the SV40 NLS var4 shown in Supplementary Fig. 2a. (**c**) Quantification of relative nuclear localization of the indicated mCherry–LINuS constructs for a population of cells before and after blue light induction. Data represent mean ± s.d. (n ≥ 5 cells, 3 independent experiments). no NLS control, mCherry fused to *As*LOV2 truncated at residue 540.

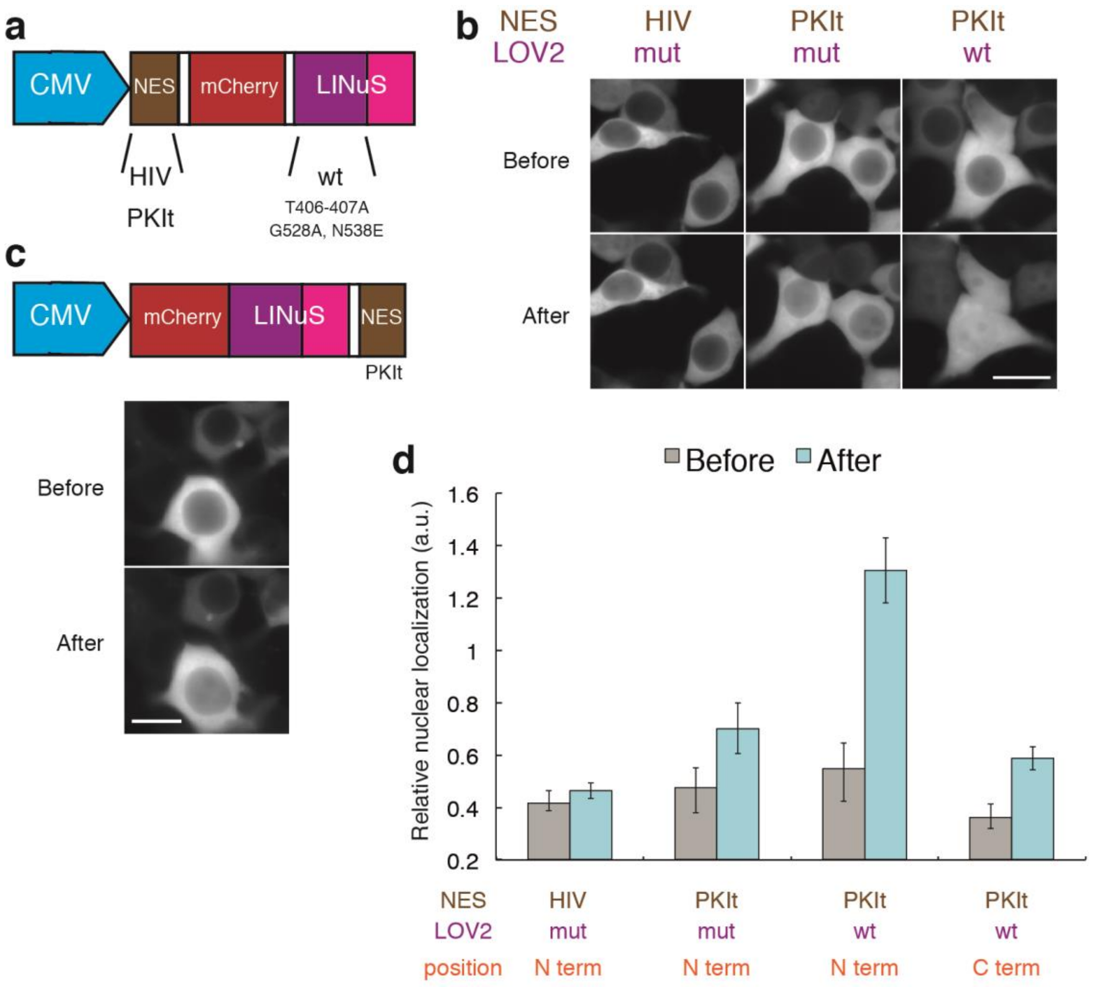

Supplementary Figure 5: Addition of an NES reduces nuclear levels in the dark state. (**a**) Schematic of mCherry-LINuS (c-Myc^{P1A}) constructs with the indicated N-terminal NESs. Either wild type or strongly caged *As*LOV2 domains were used. (**b**) Representative fluorescence microscopy images of the indicated constructs transiently transfected in HEK 293T cells before and after constant blue light illumination for 20 min. Scale bar, 20 μm. (**c**) Schematic (upper panel) and localization in HEK 293T cells (lower panel) of mCherry-LINuS (c-Myc^{P1A}) with a C-terminal PKIt NES. The white space represents a GS linker. Illumination was performed with constant blue light for 20 min. Scale bar, 15 μm. (**d**) Quantification of the relative nuclear localization of the indicated constructs before and after blue light induction. Data represent mean ± s.d. (n ≥ 6 cells, 3 independent experiments).

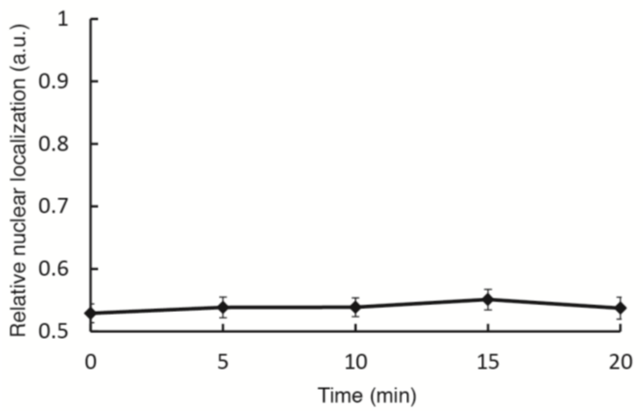

Supplementary Figure 6: mCherry-*As*LOV2 bearing the wild type Jα helix does not translocate into the nucleus upon light induction. HEK 293T cells were transiently transfected with a mCherry-*As*LOV2 fusion bearing an N-terminal PKIt NES, but no NLS and induced with 1 sec blue light pulses every 30 sec for 20 min. Data represent the mean ± s.e.m. (n = 20 cells, 2 independent experiments).

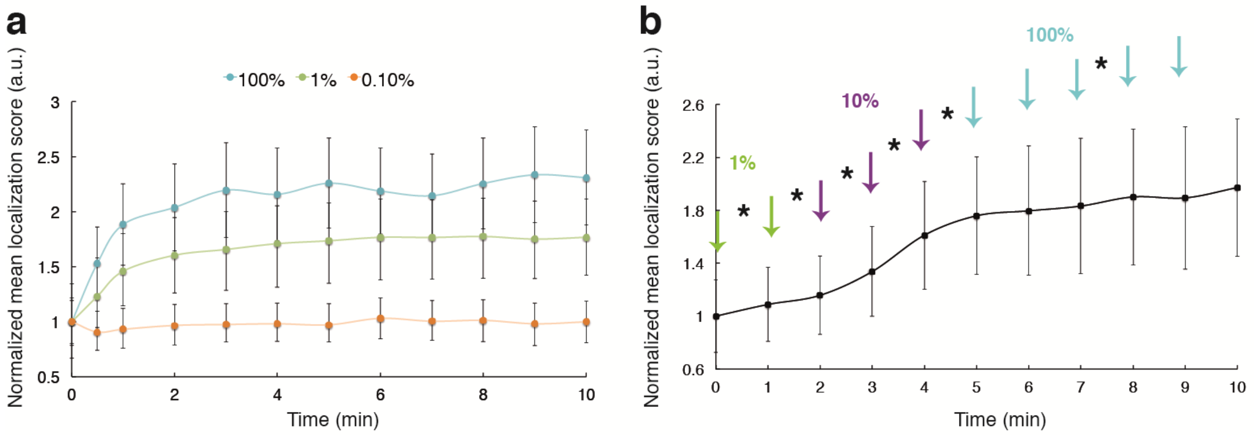

Supplementary Figure 7: Tunability of LINuS by different light intensities in *S. cerevisiae* (**a**) Graph showing the relative nuclear localization of mCherry-LINuS over time, normalized to the value before illumination (t =0). Yeast cells were illuminated with 6 sec pulses of the indicated light intensities every min for 10 min. Data represent mean ± s.d. (n = 35 cells). (**b**) Graph showing the relative nuclear localization of mCherry-LINuS over time, normalized to the value before illumination (t =0). Yeast cells were illuminated with 3 sec light pulses of increasing (indicated) intensities every min for 10 min. Data represent mean ± s.d. (n = 41 cells).

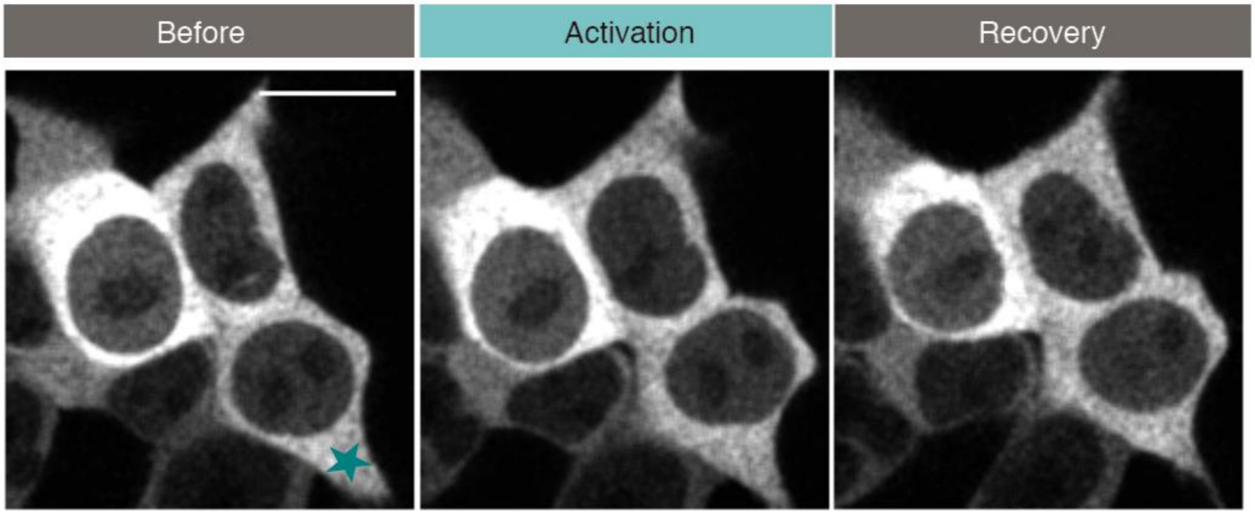

Supplementary Figure 8: mCherry-LINuS containing an impaired NLS does not translocate into the nucleus of an individually illuminated cell. Representative fluorescence time-lapse images of HEK 293T cells transfected with mCherry-LINuS bearing a mutated NLS impaired in importin binding (c-Myc^{P1AK4A}). The indicated cell (blue asterisk) was illuminated with a blue (458 nm multiline argon) laser beam directed to a confined area in the cytoplasm. Light induction was performed by scanning the region of interest for ~ 30 msec every 30 sec for 20 min followed by 20 min of recovery. Scale bar, 15 µm.

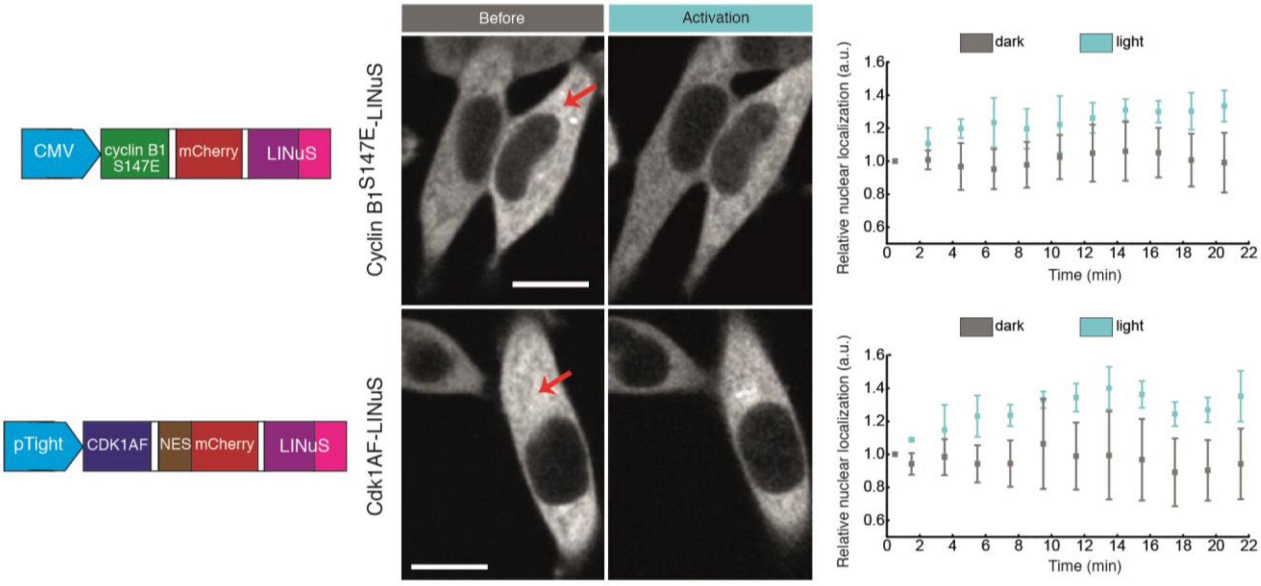

Supplementary Figure 9: Cyclin B1^{S147E} and CDK1AF nuclear translocation in single cells. Left panel, Schematic of the fusion expression constructs. CMV, strong constitutive promoter. pTight, doxycycline inducible promoter. White spaces represent glycine-serine linkers. NES, PKIt NES. LINuS indicates the *As*LOV2-NLS chimera. The NLS in these constructs is the c-Myc^{P1A}. Middle panel, representative fluorescence microscopy images of two HeLa TetON cells transiently transfected with the indicated construct. The indicated cell was illuminated with a blue (488 nm multiline argon) laser beam directed to a confined area in the cytoplasm (red arrow). Light induction was performed by scanning the region of interest for ~ 60 msec every 30 sec. CDK1AF-mCherry-LINuS expression was induced with doxycycline (1 µg/ml) for 4-5 h. Right panel, Corresponding quantification of relative nuclear localization over time, normalized to the value before activation (t =0). Data represent the mean ± s.d. At least 4 cells were analyzed for each condition. Scale bar, 15 µm.

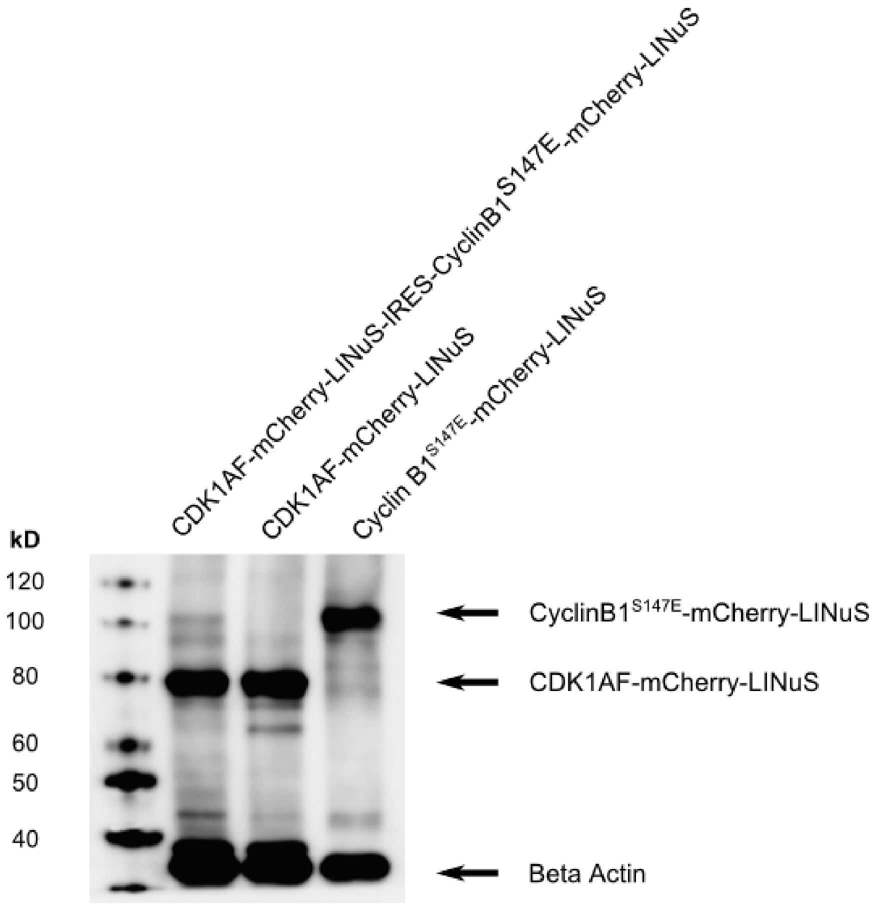

Supplementary Figure 10: Western Blot analysis of Cyclin B1^{S147E} and CDK1AF expression from a bicistronic construct. HeLa TetON cells were transiently transfected with the indicated construct. 24 h post transfection, expression was induced by doxycycline (1 µg/ml) for 5 h. Cells were lysed in lysis buffer for 10 minutes on ice and proteins were separated by SDS-page and transferred onto a nitrocellulose membrane. Subsequently, anti-mCherry antibody was applied for detecting the fusion proteins. Actin was used as loading control. First lane, MagicMark XP Western Protein Standard (Life Technologies).

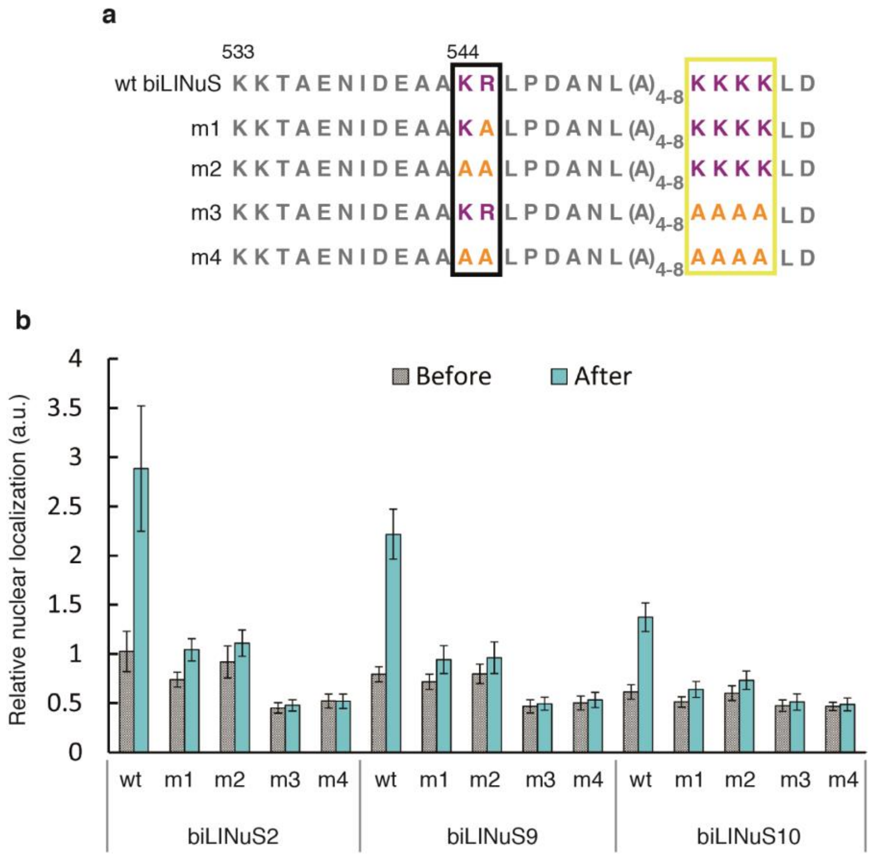

Supplementary Figure 11: Mutagenesis analysis confirms the bipartite nature of biLINuS. (**a**) Sequences of the mutants generated to test the bipartite nature of biLINuS constructs. Amino acids that surround the NLS are shown in grey. Amino acids in violet represent those present in the wild type biLINuS construct. Amino acids in orange represent mutated amino acids. Black and yellow boxes indicate the position of the first and second basic stretch (as in Fig. 4). (**b**) Quantification of the relative nuclear localization of the indicated mCherry-biLINuS variants in HEK 293T cells prior to activation (Before) and after 20 min of illumination with 1 sec blue light pulses every 30 sec for 20 min (After). Data represent the mean ± s.d. (n = 20 cells, 2 independent experiments).

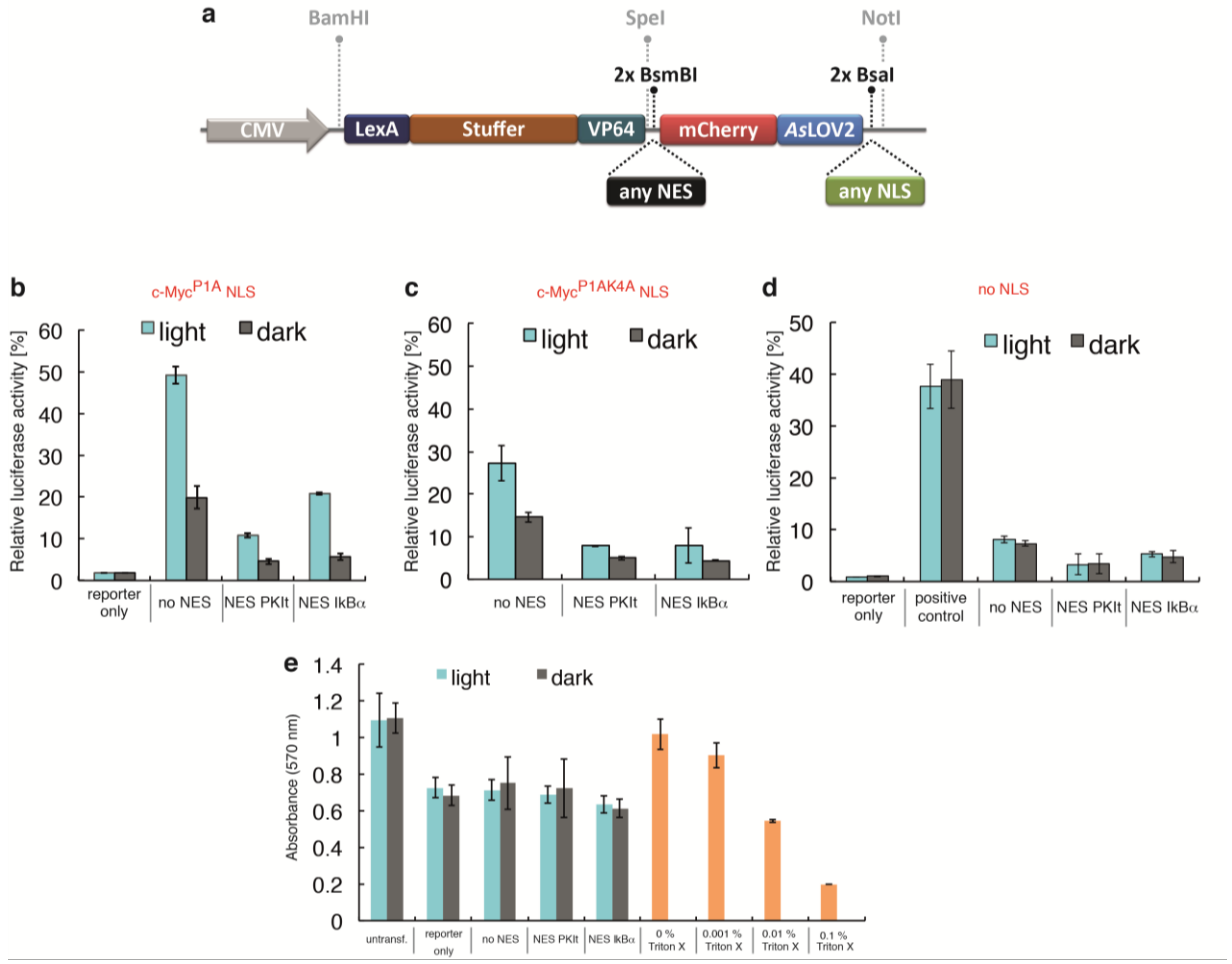

Supplementary Figure 12: Light-induced gene expression mediated by monopartite LINuS. (**a**) Schematic of a modular expression construct for a LINuS-based synthetic mammalian transcription factor. It consists of a CMV promoter, a LexA DNA binding domain (LexA), a bacterial maltose binding protein stuffer fragment (Stuffer) and a VP64 transactivation domain fused to mCherry-*As*LOV2 (truncated behind residue I539). *BsmB*I and *Bsa*I restriction sites enable the introduction of any NES or NLS sequence as annealed oligos, respectively. Unique *BamH*I, *Spe*I and *Not*I restriction sites enable the exchange of the LexA-Stuffer-VP64 or the created NES-mCherry-*As*LOV2-NLS fragments. (**b-**

d) Quantification of luciferase activity in HEK 293T cells transiently co-transfected with different transcription factor variants carrying the c-Myc^{P1A} NLS (**b**), a c-Myc^{P1AK4A} NLS mutant impaired in importin binding (**c**), or the wild type Jα helix (**d**) as well as the indicated NES, a reporter construct consisting of the Firefly luciferase gene driven from a minimal promoter coupled to four LexA binding sites, and a constitutive *Renilla* expression construct. Illumination started 15 h post transfection and was performed for 24 h with constant blue light (λ_{max} = 460 nm, ~ 10 µmol/m^2·s light intensity). Firefly luciferase activity was normalized to *Renilla* luciferase in each sample. Data represent mean ± s.d. (2 independent experiments). (**d**) Positive control, TF with the c-Myc^{P1A} NLS and no NES, locked in the pseudolit state[2]. (**e**) MTT assay for quantification of cell viability and proliferation in illuminated HEK 293T. Cells were transiently transfected as in (**b)** and illuminated for 24 h with constant blue light (λ_{max} = 460 nm, ~ 10 µmol/m^2·s light intensity). Following light induction, MTT was added to the cells for 4 h in order to allow reduction to the violet formazan. Subsequently, formazan crystals were dissolved and absorbance at 570 nm was measured in a plate reader. Non-transfected cells served as negative control. Non-transfected, non-illuminated cells treated with the indicated amounts of Triton X served as control for assay sensitivity. Note, that the Lipofectamine 2000 used for transfection in (**b,c,e)** is slightly toxic affecting the viability of transfected samples. Data represent the mean ± s.d. (4 replicates).

Supplementary tables

Supplementary Table 1 List of plasmids used in this study

Name	Backbone	Insert	Promoter	NLS	NES used	AsLOV2 variant	Source
pTriEx-PA-Rac1	pTriEx (Merck Millipore)	LOV-ipaA	CMV-chicken ß-actin hybrid	/	/	/	Ilme Schlichting (Wu *et al.* 2009)
pAA30	/	yeast codon-optimized mCherry	/	/	/	/	Alexander Anders, University of Heidelberg, Germany
pSB1A3	pSB standard backbone	/	/	/	/	/	parts.igem.org/
pcDNA3.1 (-)	Mammalian Cloning Vector	/	CMV	/	/	/	Invitrogen
p413TEF	Yest Cloning Vector	/	TEF	/	/	/	/
BBa_K165020	pSB standard backbone	LexA DNA binding domain fused to VP16 transactivator	/	/	/	/	parts.igem.org/
FKF1-VP16	pcDNA3	FKF1 DNA binding domain fused to VP64 transactivator	/	/	/	/	Masayuki Yazawa and Ricardo E Dolmetsch (Yazawa et al. 2009)
BBa_K165031	pSB standard backbone	mCYC promoter plus 4 LexA binding sites		/	/	/	iGEM partsregistry: parts.igem.org/
pFR-Luc	/	Firefly Luciferase	Gal4 dependent promoter	/	/	/	Agilent
pmCherry-N1	/	/	CMV	/	/	/	Clontech
pRL-TK	/	*renilla* luciferase	TK	/	/	/	Promega
CyclinB1	p1055	Cyclin B1-FRB-YFP	/	/	/	/	James E. Ferrell (Santos et al. 2012)
CDK1AF	pTreTight	constitutively active $CDK1^{T14A,Y15F}$	pTight (TRE + $_{min}$CMVΔ)	/	/	/	James E. Ferrell (Santos et al. 2012)
pGal4-eYFP	pDN	eYFP	Gal4 dependent promoter	/	/	/	/
pCMV-YFP-IRES-neo	/	YFP-IRES-neomycin	CMV	/	/	/	Dirk Grimm, University of Heidelberg, Germany
pDB1	pTEF413	mCherry fused to *As*LOV2 tr. 2*	TEF	not present	not present	N538E	This Work
pDB2	pTEF413	mCherry fused to LINuS	TEF	SV40, tr. 2*	not present	N538E	This Work
pDB3	pTEF413	mCherry fused to LINuS	TEF	SV40, tr. 1*	not present	N538E	This Work
pDB4	pTEF413	mCherry fused to LINuS	TEF	$SV40^{P1A}$, tr. 2*	not present	N538E	This Work
**pDB5**	**pTEF413**	**mCherry fused to LINuS**	**TEF**	**$SV40^{P1A}$, tr. 1***	**not present**	**N538E**	**This Work**
pDB6	pcDNA3.1 (-)	mCherry fused to *As*LOV2 tr. 2*	CMV	not present	not present	N538E	This Work

pDB7	pcDNA3.1 (-)	mCherry fused to LINuS	CMV	SV40, tr. 2*	not present	N538E	This Work
pDB8	pcDNA3.1 (-)	mCherry fused to LINuS	CMV	SV40, tr. 1*	not present	N538E	This Work
pDB9	pcDNA3.1 (-)	mCherry fused to LINuS	CMV	SV40^{P1A}, tr. 2*	not present	N538E	This Work
pDB10	pcDNA3.1 (-)	mCherry fused to LINuS	CMV	SV40^{P1A}, tr. 1*	not present	N538E	This Work
pDB11	pcDNA3.1 (-)	mCherry fused to LINuS	CMV	SV40^{K4A}	not present	wt	This Work
pDB12	pcDNA3.1 (-)	mCherry fused to LINuS	CMV	c-Myc	not present	wt	This Work
pDB13	pcDNA3.1 (-)	mCherry fused to LINuS	CMV	c-Myc^{P1A}	not present	wt	This Work
pDB14	pcDNA3.1 (-)	mCherry fused to LINuS	CMV	E1A$_{pent}$	not present	wt	This Work
pDB15	pcDNA3.1 (-)	mCherry fused to LINuS	CMV	VP3	not present	wt	This Work
pDB16	pcDNA3.1 (-)	mCherry fused to LINuS	CMV	SV40^{P1A}, tr. 1*	not present	T406-407A, N538E	This Work
pDB17	pcDNA3.1 (-)	mCherry fused to LINuS	CMV	SV40^{P1A}, tr. 1*	not present	T406-407A, G528A, N538E	This Work
pDB18	pcDNA3.1 (-)	mCherry fused to LINuS	CMV	c-Myc^{P1A}	not present	T406-407A, G528A	This Work
pDB19	pcDNA3.1 (-)	mCherry fused to LINuS	CMV	c-Myc^{P1A}	not present	T406-407A, G528A, N538E	This Work
pDB20	pcDNA3.1 (-)	mCherry fused to LINuS	CMV	c-Myc^{P1A}	not present	T406-407A, I532A, G528A, N538E	This Work
pDB21	pcDNA3.1 (-)	mCherry fused to LINuS	CMV	c-Myc^{P1A}	HIV (N-terminal)	T406-407A, G528A, N538E	This Work
**pDB22**	**pcDNA3.1 (-)**	**mCherry fused to LINuS**	**CMV**	**c-Myc^{P1A}**	**PKIt (N-terminal)**	**wt**	**This Work**
pDB23	pcDNA3.1 (-)	mCherry fused to LINuS	CMV	c-Myc^{P1A}	PKIt (N-terminal)	T406-407A, G528A, N538E	This Work
pDB24	pcDNA3.1 (-)	mCherry fused to LINuS	CMV	c-Myc^{P1A}	PKIt (C-terminal)	wt	This Work
pJR25	pcDNA3.1 (-)	mCherry fused to LINuS bearing mutated NLS	CMV	c-Myc^{P1AK4A}	PKIt (N-terminal)	wt	This Work
pDN26	pcDNA3.1 (-)	mCherry fused to wt *As*LOV2	CMV	not present	PKIt (N-terminal)	wt	This Work
pDB27	pcDNA3.1 (-)	LINuS fused to mCherry	CMV	c-Myc^{P1A}	not present	wt	This Work
pTD28	pTreTight	constitutively active CDK1^{T14A,Y15F} fused to mChery-LINuS	pTight (TRE + $_{min}$CMVΔ)	c-Myc^{P1A}	PKIt	wt	This Work
pDN29	pTreTight	constitutively active CDK1^{T14A,Y15F} fused to eYFP-LINuS	pTight (TRE + $_{min}$CMVΔ)	c-Myc^{P1A}	PKIt	wt	This Work
pTD30	pmCherry-N1	Cyclin B1^{S147E} fused to mCherry-LInuS	CMV	c-Myc^{P1A}	not present	wt	This Work
pDN31	pTreTight	Cyclin B1^{S147E} fused to mCherry-LInuS	CMV	c-Myc^{P1A}	not present	wt	This Work
**pJR32**	**pTreTight**	**CDK1(T14A,Y15F) - mCherry- LINuS-IRES-CyclinB1(S147E)-mCherry-LINuS**	**pTight (TRE + $_{min}$CMVΔ)**	**c-Myc^{P1A}**	**PKIt (N-terminal of CDK1)**	**wt**	**This Work**

pDN33	pcDNA3.1 (-)	mCherry fused to biLINuS	CMV	biNLS1	PKIt (N-terminal)	wt	This Work
**pDN34**	**pcDNA3.1 (-)**	**mCherry fused to biLINuS**	**CMV**	**biNLS2**	**PKIt (N-terminal)**	**wt**	**This Work**
pDN35	pcDNA3.1 (-)	mCherry fused to biLINuS	CMV	biNLS3	PKIt (N-terminal)	wt	This Work
pDN36	pcDNA3.1 (-)	mCherry fused to biLINuS	CMV	biNLS4	PKIt (N-terminal)	wt	This Work
pDN37	pcDNA3.1 (-)	mCherry fused to biLINuS	CMV	biNLS5	PKIt (N-terminal)	wt	This Work
pDN38	pcDNA3.1 (-)	mCherry fused to biLINuS	CMV	biNLS6	PKIt (N-terminal)	wt	This Work
pDN39	pcDNA3.1 (-)	mCherry fused to biLINuS	CMV	biNLS7	PKIt (N-terminal)	wt	This Work
pDN40	pcDNA3.1 (-)	mCherry fused to biLINuS	CMV	biNLS8	PKIt (N-terminal)	wt	This Work
**pDN41**	**pcDNA3.1 (-)**	**mCherry fused to biLINuS**	**CMV**	**biNLS9**	**PKIt (N-terminal)**	**wt**	**This Work**
**pDN42**	**pcDNA3.1 (-)**	**mCherry fused to biLINuS**	**CMV**	**biNLS10**	**PKIt (N-terminal)**	**wt**	**This Work**
**pDN43**	**pcDNA3.1 (-)**	**mCherry fused to biLINuS**	**CMV**	**biNLS11**	**PKIt (N-terminal)**	**wt**	**This Work**
pDN44	pcDNA3.1 (-)	mCherry fused to biLINuS	CMV	biNLS12	PKIt (N-terminal)	wt	This Work
pDN45	pcDNA3.1 (-)	mCherry fused to biLINuS	CMV	biNLS13	PKIt (N-terminal)	wt	This Work
pDN46	pcDNA3.1 (-)	mCherry fused to biLINuS	CMV	biNLS14	PKIt (N-terminal)	wt	This Work
pDN47	pcDNA3.1 (-)	mCherry fused to biLINuS	CMV	biNLS15	PKIt (N-terminal)	wt	This Work
pDN48	pcDNA3.1 (-)	mCherry fused to biLINuS	CMV	biNLS16	PKIt (N-terminal)	wt	This Work
pDN49	pcDNA3.1 (-)	mCherry fused to biLINuS	CMV	biNLS17	PKIt (N-terminal)	wt	This Work
pDN50	pcDNA3.1 (-)	mCherry fused to biLINuS	CMV	biNLS18	PKIt (N-terminal)	wt	This Work
pDN51	pcDNA3.1 (-)	mCherry fused to biLINuS	CMV	biNLS19	PKIt (N-terminal)	wt	This Work
pDN52	pcDNA3.1 (-)	mCherry fused to biLINuS	CMV	biNLS20	PKIt (N-terminal)	wt	This Work
pDN53	pcDNA3.1 (-)	mCherry fused to biLINuS	CMV	biNLS21	PKIt (N-terminal)	wt	This Work
**pDN54**	**pcDNA3.1 (-)**	**mCherry fused to biLINuS**	**CMV**	**biNLS22**	**PKIt (N-terminal)**	**wt**	**This Work**
pDN55	pcDNA3.1 (-)	mCherry fused to biLINuS	CMV	biNLS23	PKIt (N-terminal)	wt	This Work
pDN56	pcDNA3.1 (-)	mCherry fused to biLINuS	CMV	biNLS24	PKIt (N-terminal)	wt	This Work
pDN57	pcDNA3.1 (-)	mCherry fused to biLINuS	CMV	biNLS25	PKIt (N-terminal)	wt	This Work
pDN58	pcDNA3.1 (-)	mCherry fused to biLINuS	CMV	biNLS26	PKIt (N-terminal)	wt	This Work
pDN59	pcDNA3.1 (-)	mCherry fused to biLINuS	CMV	biNLS27	PKIt (N-terminal)	wt	This Work
pDN60	pcDNA3.1 (-)	mCherry fused to biLINuS	CMV	biNLS28	PKIt (N-terminal)	wt	This Work
pDN61	pcDNA3.1 (-)	mCherry fused to biLINuS	CMV	biNLS29	PKIt (N-terminal)	wt	This Work
pDN62	pcDNA3.1 (-)	mCherry fused to biLINuS	CMV	biNLS30	PKIt (N-terminal)	wt	This Work
pDN63	pcDNA3.1 (-)	mCherry fused to biLINuS	CMV	biNLS2 m1	PKIt (N-terminal)	wt	This Work
pDN64	pcDNA3.1 (-)	mCherry fused to biLINuS	CMV	biNLS2 m2	PKIt (N-terminal)	wt	This Work
pDN65	pcDNA3.1 (-)	mCherry fused to biLINuS	CMV	biNLS2 m3	PKIt (N-terminal)	wt	This Work

pDN66	pcDNA3.1 (-)	mCherry fused to biLINuS	CMV	biNLS2 m4	PKIt (N-terminal)	wt	This Work
pDN67	pcDNA3.1 (-)	mCherry fused to biLINuS	CMV	biNLS9 m1	PKIt (N-terminal)	wt	This Work
pDN68	pcDNA3.1 (-)	mCherry fused to biLINuS	CMV	biNLS9 m2	PKIt (N-terminal)	wt	This Work
pDN69	pcDNA3.1 (-)	mCherry fused to biLINuS	CMV	biNLS9 m3	PKIt (N-terminal)	wt	This Work
pDN70	pcDNA3.1 (-)	mCherry fused to biLINuS	CMV	biNLS9 m4	PKIt (N-terminal)	wt	This Work
pDN71	pcDNA3.1 (-)	mCherry fused to biLINuS	CMV	biNLS10 m1	PKIt (N-terminal)	wt	This Work
pDN72	pcDNA3.1 (-)	mCherry fused to biLINuS	CMV	biNLS10 m2	PKIt (N-terminal)	wt	This Work
pDN73	pcDNA3.1 (-)	mCherry fused to biLINuS	CMV	biNLS10 m3	PKIt (N-terminal)	wt	This Work
pDN74	pcDNA3.1 (-)	mCherry fused to biLINuS	CMV	biNLS10 m4	PKIt (N-terminal)	wt	This Work
pDN75	pcDNA3.1 (-)	mCherry fused to biLINuS	CMV	biNLS2	HIV (N-terminal)	wt	This Work
**pDN76**	**pcDNA3.1 (-)**	**mCherry fused to biLINuS**	**CMV**	**biNLS2**	**HIVt (N-terminal)**	**wt**	**This Work**
**pDN77**	**pcDNA3.1 (-)**	**mCherry fused to biLINuS**	**CMV**	**biNLS2**	**IkBα (N-terminal)**	**wt**	**This Work**
pDN78	pcDNA3.1 (-)	mCherry fused to biLINuS	CMV	biNLS2	double PKIt (N-terminal)	wt	This Work
pDN79	pcDNA3.1 (-)	mCherry fused to biLINuS	CMV	biNLS2	MAPKK (N-terminal)	wt	This Work
**pDN80**	**pmCherry-N1**	**LINuS-flex: modular LINuS construct enabling introduction of any protein encoding sequence, NES and NLS**	**CMV**	**not present**	**not present**	**wt**	**This Work**
pDN81	pmCherry-N1	LexA-VP64 transcription factor fused to mCherry-LINuS	CMV	c-Myc^{P1A}	not present	wt	This Work
pDN82	pmCherry-N1	LexA-VP64 transcription factor fused to mCherry-LINuS	CMV	c-Myc^{P1A}	PKIt	wt	This Work
pDN83	pmCherry-N1	LexA-VP64 transcription factor fused to mCherry-LINuS	CMV	c-Myc^{P1A}	IkBα	wt	This Work
pDN84	pmCherry-N1	LexA-VP64 transcription factor fused to mCherry-AsLOV2	CMV	not present	not present	wt	This Work
pDN85	pmCherry-N1	LexA-VP64 transcription factor fused to mCherry-AsLOV2	CMV	not present	PKIt	wt	This Work
pDN86	pmCherry-N1	LexA-VP64 transcription factor fused to mCherry-AsLOV2	CMV	not present	IkBα	wt	This Work
pDN87	pmCherry-N1	LexA-VP64 transcription factor fused to mCherry-LINuS bearing mutated NLS	CMV	c-Myc^{P1AK4A}	not present	wt	This Work

pDN88	pmCherry-N1	LexA-VP64 transcription factor fused to mCherry-LINuS bearing mutated NLS	CMV	c-Myc^{P1AK4A}	PKIt	wt	This Work
pDN89	pmCherry-N1	LexA-VP64 transcription factor fused to mCherry-LINuS bearing mutated NLS	CMV	c-Myc^{P1AK4A}	IkBα	wt	This Work
**pDN90**	**pmCherry-N1**	**LexA-VP64 transcription factor fused to mCherry-LINuS**	**CMV**	**biNLS2**	**not present**	**wt**	**This Work**
**pDN91**	**pmCherry-N1**	**LexA-VP64 transcription factor fused to mCherry-LINuS**	**CMV**	**biNLS2**	**PKIt**	**wt**	**This Work**
**pDN92**	**pmCherry-N1**	**LexA-VP64 transcription factor fused to mCherry-LINuS**	**CMV**	**biNLS2**	**IkBα**	**wt**	**This Work**
**pDN93**	**pmCherry-N1**	**LexA-VP64 transcription factor fused to mCherry-LINuS**	**CMV**	**biNLS9**	**not present**	**wt**	**This Work**
**pDN94**	**pmCherry-N1**	**LexA-VP64 transcription factor fused to mCherry-LINuS**	**CMV**	**biNLS9**	**PKIt**	**wt**	**This Work**
**pDN95**	**pmCherry-N1**	**LexA-VP64 transcription factor fused to mCherry-LINuS**	**CMV**	**biNLS9**	**IkBα**	**wt**	**This Work**
**pDN96**	**pmCherry-N1**	**LexA-VP64 transcription factor fused to mCherry-LINuS**	**CMV**	**biNLS10**	**not present**	**wt**	**This Work**
**pDN97**	**pmCherry-N1**	**LexA-VP64 transcription factor fused to mCherry-LINuS**	**CMV**	**biNLS10**	**PKIt**	**wt**	**This Work**
**pDN98**	**pmCherry-N1**	**LexA-VP64 transcription factor fused to mCherry-LINuS**	**CMV**	**biNLS10**	**IkBα**	**wt**	**This Work**
pDN99	pmCherry-N1	LexA-VP64 transcription factor fused to mCherry-LINuS	CMV	c-Myc^{P1A}	not present	I539E	This Work
**pDN100**	**pFR-Luc**	**firefly luciferase**	**4xLexA binding site dependent minimal promoter**	**/**	**/**	**/**	**This Work**

*tr. 1 and tr. 2 indicate truncation of the *As*LOV2 domain at residues 539 or 540 respectively. Bold entries indicate best performing constructs.

Supplementary Table 2 List of oligonucleotide sequences

Number	Sequence 5' --> 3'	NLS or NES inroduced	introduced mutation
1	tttt**tgtaca**agggtggatctggaggttcaggtggaagtttggctgccgctcttgaacgtattgag		
2	tttt**gcggccgc**ctagacctttctcttctttttggatcaatctcttctgcagttttcttaatc	SV40 variant 1	AsLOV2 N538E
3	tttt**gcggccgc**ctagacctttctcttctttttggaatctcttctgcagttttcttaatcag	SV40 variant 2	AsLOV2 N538E
4	tttt**gcggccgc**ctagacctttctcttcttttggcatcaatctcttctgcagttttcttaatc	SV40 variant 3	AsLOV2 N538E
5	tttt**gcggccgc**ctagacctttctcttcttttggcaatctcttctgcagttttcttaatcag	SV40 variant 4	AsLOV2 N538E
6	tttt**gcggccgc**ctaatcaatctcttctgcagttttcttaatc	no NLS (wt Jα helix)	
7	cctt**gaattc**gcggccgcttctagacgtacgctgcaggtcg	/	
8	aagg**actagt**ctaaatcgatgagttcgagctcg	/	
9	tttt**ctcgag**ctgcagcggccgccta	/	
10	tttt**ctcgag**ctagtccaacttgaccctcttggcagcaggaatattttctgcagttttcttaatcag	c-Myc NLS	
11	tttt**ctcgag**ctagtccaacttgaccctcttggcagcggcaatattttctgcagttttcttaatcag	c-Myc^{P1A} NLS	
12	tttt**ctcgag**ctaaggcctaggtctcttatcaatattttctgcagttttctt	E1A NLS	
13	tttt**ctcgag**ctactttctcttctttttatcaatattttctgcagttttctt	VP3pent NLS	
14	tttt**ctcgag**ctagacctttctggcctttttggaatattttctgcagttttcttaatcag	SV40^{K4A} NLS	
15	ttttatcaatattttctgcagttttcttagccagcatgacggcctc	/	AsLOV2 G528A
16	ttttatcaatattttctgcagttttcttaatcagcatgacggcctc	/	AsLOV2 I532A/G528A
17	tttt**tgtaca**agggtggatctggaggttcaggtggaagtttggctgccgctcttgaacgtattgag	/	AsLOV2 T406-7A
18	tttt**ctcgag**ctagtccaacttgaccctcttggcagcggcaatctcttctgcagttttct	c-Myc^{P1A} NLS	AsLOV2 N538E
19	ttt**gctagc**atgttagccttgaaattagcaggtcttgatatcgtgagcaagggcgaggag	PKIt NES	
20	ttt**gctagc**atgcttcaacttcctcctcttgagagacttactcttgtgagcaagggcgaggag	HIV NES	
21	ttt**ctcgag**ctagatatcaagacctgctaatttcaaggctaagctgccgccgctgccgccgctgccgccgtccaacttgacc ctcttg	c-Myc^{P1A} NLS -GS linker- PKIt NES	
22	tttt**ctcgag**tctagagggcccgtttaaacccgctgatc	c-Myc^{P1AK4A} NLS	c-Myc P1A4KA
23	aaaa**ctcgag**ctagtccaacttgaccctcgcggcagcggcaatattttc	c-Myc^{P1AK4A} NLS	c-Myc P1A4KA
24	tttt**gctagc**atgttggctactacacttgaacg	/	
25	tttt**gaattc**gtccaacttgaccctcttg	/	
26	tttt**gaattc**ggtggatctggaggttcaggtggaagtcgtacgctgcaggtcg	/	
27	ttttt**ctcgag**ctaaatcgatgagttcgagctcg	/	
28	tttt**gcggccgc**aggtggatctggaggttcaggtggaagtatgttagccttgaaattagcaggtcttgatatcg	PKIT NES	
29	tttt**aagctt**ctagtccaacttgaccctcttggcagcggc	c-Myc^{P1A} NLS	
30	tttt**cgtctc**ggatccgccaccatggcgctccgagtcaccaggaac	/	
31	tttt**cgtctc**aatccagatgtttccattgggcttggagaggcagtatcaaccaaaataggctc	/	
32	tttt**cgtctc**aggatgtgcccctgcagaagaagacctgtgtcaggctttcgaggatgtaattcttgcagtaaatgatgtg	/	Cyclin B1 S147E
33	tttt**cgtctc**actagtacttccacctgaacctccagatc	/	
34	tttt**gtcgac**gcccctctccctcccccccccctaac	/	
35	tttt**ggatcc**tttttcaaaggaaaaccacgtcccc	/	
36	tttt**cgtctc**tcaatattttctgcagttttcttaatcAG	/	
37	tttt**cgtctc**ttagctcgagcggccgccactgtgctggatatc	/	
38	attgacgaggccgcaaagagactgcccgacgccaacctggccgcacccaagaccaagagaaaggtg	biNLS1	
39	gctacacctttctcttggtcttgggtgcggccaggttggcgtcgggcagtctctttgcggcctcgt		
40	attgacgaggccgcaaagagactgcccgacgccaacctggcagccgcagccaagaagaaaaagctggac	biNLS2	
41	gctagtccagctttttcttcttggctgcggctgccaggttggcgtcgggcagtctctttgcggcctcgt		
42	attgacgaggccgcaaaggagctgcccgccgcaaagaagaagaaactggac	biNLS3	
43	gctagtccagtttcttcttctttgcggcgggcagctcctttgcggcctcgt		
44	attgaccccgccgcaaagaagaagaaactggacgccaacctg	biNLS4	
45	gctacaggttggcgtccagtttcttcttctttgcggcggggt		
46	attgaccccgccgcaaagaagaagaaactggacgccaacctgttc	biNLS5	
47	gctagaacaggttggcgtccagtttcttcttctttgcggcggggt		
48	attgacgaggccgcaaagagactgcccgacgccaacctggccgcagccaagaagaaaaagctggac	biNLS6	
49	gctagtccagctttttcttcttggctgcggccaggttggcgtcgggcagtctctttgcggcctcgt		
50	attgacgaggccgcaaagagactgcccgacgccaacctggccgcaaagaagaaaaagctggac	biNLS7	
51	gctagtccagctttttcttctttgcggccaggttggcgtcgggcagtctctttgcggcctcgt		
52	attgacgaggccgcaaagagactgcccgacgccaacctggccaagaagaaaaagctggac	biNLS8	
53	gctagtccagctttttcttcttggccaggttggcgtcgggcagtctctttgcggcctcgt		
54	attgacgaggccgcaaagagactgcccgacgccaacctggccgcagcagccgcagccaagaagaaaaagctggac	biNLS9	
55	gctagtccagctttttcttcttggctgcggctgctgcggccaggttggcgtcgggcagtctctttgcggcctcgt		
56	attgacgaggccgcaaagagactgcccgacgccaacctggccgcagcagccgcagccgcagcaaagaagaaaaagct ggac	biNLS10	
57	gctagtccagctttttcttctttgctgcggctgcggctgctgcggccaggttggcgtcgggcagtctctttgcggcctcgt		

58	attgacgaggccgcaaagagactgcccgacgccaacctggcagccgcagccaagaccaagagaaagaaactggac	biNLS11	
59	gctagtccagtttctttctcttggtcttggctgcggctgccaggttggcgtcgggcagtctctttgcggcctcgt		
60	attgacgaggccaagagactgcccgacgccaacctggcagccgcagccaagaagaaaaagctggac	biNLS12	
61	gctagtccagctttttcttcttggctgcggctgccaggttggcgtcgggcagtctcttggcctcgt		
62	attgacgagaagagactgcccgacgccaacctggcagccgcagccaagaagaaaaagctggac	biNLS13	
63	gctagtccagctttttcttcttggctgcggctgccaggttggcgtcgggcagtctcttctcgt		
64	attgacaagagactgcccgacgccaacctggcagccgcagccaagaagaaaaagctggac	biNLS14	
65	gctagtccagctttttcttcttggctgcggctgccaggttggcgtcgggcagtctcttgt		
66	attgacgaggcacgcaaggagctgcccgacgcaaacctggccgcagccaagaagaaaaagctggac	biNLS15	
67	gctagtccagctttttcttcttggctgcggccaggtttgcgtcgggcagctccttgcgtgcctcgt		
68	attgacgaggccaagaaggagctgcccgacgccaacctggccgcagcaaagaagaaaaagctggac	biNLS16	
69	gctagtccagctttttcttctttgctgcggccaggttggcgtcgggcagctccttcttggcctcgt		
70	attgacgaggcccgcaaggagctgcccgacgccaacctggcagcagcccgcaagaccaagaaaaagataaagggcatccagcaggccaccgca	biNLS17	
71	gctatgcggtggcctgctggatgccctttatctttttcttggtcttgcgggctgctgccaggttggcgtcgggcagctccttgcgggcctcgt		
72	attgacgaggccaagaaggagctgcccgacgcaaacctggcagcagcccgccgccgcaacctg	biNLS18	
73	gctacaggttgcggcggcgggctgctgccaggtttgcgtcgggcagctccttcttggcctcgt		
74	attgacgaggccgcaaaggagctgcgccgccgc	biNLS19	
75	gctagcggcggcgcagctcctttgcggcctcgt		
76	attgacgaggcagccaaggagctgcccgacgcaaacctgcgccgccgc	biNLS20	
77	gcggcggcgcaggtttgcgtcgggcagctccttggctgcctcgt		
78	attgacgaggcagccaaggagctgcgccgccgcaacctg	biNLS21	
79	gctacaggttgcggcggcgcagctccttggctgcctcgt		
80	attgacgaggccgcaaagagactgcccgacgccaacctggccgcagcagccgcagccgcaaagaagaaaaagctggac	biNLS22	
81	gctagtccagctttttcttctttgcggctgcggctgctgcggccaggttggcgtcgggcagtctctttgcggcctcgt		
82	attgacgaggcagccaaggagctgaagaagaaaaagctggac	biNLS23	
83	gctagtccagctttttcttcttcagctccttggctgcctcgt		
84	attgacgaggcagccaaggagctgcccaagaagaaaaagctggac	biNLS24	
85	gctagtccagctttttcttcttgggcagctccttggctgcctcgt		
86	attgacgaggcagccaaggagctgcccgacgcaaagaagaaaaagctggac	biNLS25	
87	gctagtccagctttttcttctttgcgtcgggcagctccttggctgcctcgt		
88	attgacgaggcagccaaggagctgcccgacgcaaacctgaagaagaaaaagctggac	biNLS26	
89	gctagtccagctttttcttcttcaggtttgcgtcgggcagctccttggctgcctcgt		
90	attgacgaggccgcaaaggagctgcccgacgccaacctggccgcagcagccgcagccaagaagaaaaagctggac	biNLS27	
91	gctagtccagctttttcttcttggctgcggctgctgcggccaggttggcgtcgggcagctcctttgcggcctcgt		
92	attgacgaggcacgcaaggagctgcccgacgcaaacctggcagccgcagccaagaagaaaaagctggac	biNLS28	
93	gctagtccagctttttcttcttggctgcggctgccaggtttgcgtcgggcagctccttgcgtgcctcgt		
94	attgacgaggccaagaaggagctgcccgacgccaacctggcagccgcagccaagaagaaaaagctggac	biNLS29	
95	gctagtccagctttttcttcttggctgcggctgccaggttggcgtcgggcagctccttcttggcctcgt		
96	attgacgaggcacgcaaggagctgcccgacgcaaacctggccgcagcagccgcagccaagaagaaaaagctggac	biNLS30	
97	gctagtccagctttttcttcttggctgcggctgctgcggccaggtttgcgtcgggcagctccttgcgtgcctcgt		
98	attgacgaggcagccaaggcactgcccgacgccaacctggcagccgcagccaagaagaaaaagctggac	biNLS2 m1	
99	gctagtccagctttttcttcttggctgcggctgccaggttggcgtcgggcagtgccttggctgcctcgt		
100	attgacgaggcagccgcagccctgcccgacgccaacctggcagccgcagccaagaagaaaaagctggac	biNLS2 m2	
101	gctagtccagctttttcttcttggctgcggctgccaggttggcgtcgggcagggctgcggctgcctcgt		
102	attgacgaggccgcaaagagactgcccgacgccaacctggccgcagcagccgcagccgcagcactggac	biNLS2 m3	
103	gctagtccagtgctgcggctgcggctgctgcggccaggttggcgtcgggcagtctctttgcggcctcgt		
104	attgacgaggcagccgcagccctgcccgacgccaacctggccgcagcagccgcagccgcagcactggac	biNLS2 m4	
105	gctagtccagtgctgcggctgcggctgctgcggccaggttggcgtcgggcagggctgcggctgcctcgt		
106	attgacgaggcagccaaggcactgcccgacgccaacctggccgcagcagccgcagccaagaagaaaaagctggac	biNLS9 m1	
107	gctagtccagctttttcttcttggctgcggctgctgcggccaggttggcgtcgggcagtgccttggctgcctcgt		
108	attgacgaggcagccgcagccctgcccgacgccaacctggccgcagcagccgcagccaagaagaaaaagctggac	biNLS9 m2	
109	gctagtccagctttttcttcttggctgcggctgctgcggccaggttggcgtcgggcagggctgcggctgcctcgt		
110	attgacgaggccgcaaagagactgcccgacgccaacctggccgcagcagccgcagccgcagcagcagccctggac	biNLS9 m3	
111	gctagtccagggctgctgctgcggctgcggctgctgcggccaggttggcgtcgggcagtctctttgcggcctcgt		
112	attgacgaggcagccgcagccctgcccgacgccaacctggccgcagcagccgcagccgcagcagcagccctggac	biNLS9 m4	
113	gctagtccagggctgctgctgcggctgcggctgctgcggccaggttggcgtcgggcagggctgcggctgcctcgt		
114	attgacgaggcagccaaggcactgcccgacgccaacctggccgcagcagccgcagccgcagcaaagaagaaaaagctggac	biNLS10 m1	
115	gctagtccagctttttcttctttgctgcggctgcggctgctgcggccaggttggcgtcgggcagtgccttggctgcctcgt		
116	attgacgaggcagccgcagccctgcccgacgccaacctggccgcagcagccgcagccgcagcaaagaagaaaaagctggac	biNLS10 m2	

117	gctagtccagctttttcttctttgctgcggctgcggctgctgcggccaggttggcgtcgggcagggctgcggctgcctcgt		
118	attgacgaggccgcaaagagactgcccgacgccaacctggccgcagcagccgcagccgcagcagcagccgcagcactg gac	biNLS10 m3	
119	gctagtccagtgctgcggctgctgctgcggctgcggctgctgcggccaggttggcgtcgggcagtctctttgcggcctcgt		
120	attgacgaggcagccgcagccctgcccgacgccaacctggccgcagcagccgcagccgcagcagcagccgcagcactg gac	biNLS10 m4	
121	gctagtccagtgctgcggctgctgctgcggctgcggctgctgcggccaggttggcgtcgggcagggctgcggctgcctcgt		
122	tttt**cgtctc**tgtgagcaagggcgaggaggataac	/	
123	tttt**cgtctc**tcatgctagccagcttgggtctccctatagtg	/	
124	catgcttcaacttcctcctcttgagagacttactctt	HIV NES	
125	tcacaagagtaagtctctcaagaggaggaagttgaag		
126	catgcttcctcctcttgagagacttactctt	truncated HIV NES	
127	tcacaagagtaagtctctcaagaggaggaag		
128	catgcccagcacccggatccagcagcagctgggccagctgaccctggagaacctgcag	IkBα NES	
129	tcacctgcaggttctccagggtcagctggcccagctgctgctggatccgggtgctggg		
130	catgttagccttgaaattagcaggtcttgatatcggcggaagcggaggcagcttagccttgaaattagcaggtcttgatatc	2x PKIt NES	
131	tcacgatatcaagacctgctaatttcaaggctaagctgcctccgcttccgccgatatcaagacctgctaatttcaaggctaa		
132	catgaacctggtggacctgcagaagaagctggaggagctggagctggacgagcagcag	MAPKK NES	
133	tcacctgctgctcgtccagctccagctcctccagcttcttctgcaggtccaccaggtt		
134	tttt**ggatcc**accggtcgccaccatgaaagcgttaacggccaggcaac	/	
135	aaaa**ggtctc**agctggttccgctaccacctggttcaccggcagccacacgac	/	
136	aaaa**ggtctc**acagcaaaataaaaacaggtgcacgcatcctc	/	
137	tttt**ggtctc**agccgttatagcctttatcgccgttaatc	/	
138	aaaa**ggtctc**acggcctcgctgaagtcggtaagaaattc	/	
139	aaaa**ggtacc**gctgccgcccttggtgatacgagtctgcgcgtctttc	/	
140	tttt**cccggg**actagtggagacgccggcggcagcggcggcagcgtcgtctcagtgagcaagggcgaggaggataac	/	
141	aaaa**gcggccgc**tgagacctttaatttctactattggtctccaatattttctgcagttttcttaatcag	/	
142	tagtggcggaagcggaggcagcggcggaagcttagccttgaaattagcaggtcttgatatc	PKIt NES	
143	tcacgatatcaagacctgctaatttcaaggctaagcttccgccgctgcctccgcttccgcc		
144	tagtggcggaagcggaggcagcggcggaagccccagcacccggatccagcagcagctgggccagctgaccctggagaa cctgcag	IkBα NES	
145	tcacctgcaggttctccagggtcagctggcccagctgctgctggatccgggtgctggggcttccgccgctgcctccgcttcc gcc		
146	tattgccgccgccaagcgggtgaagctggactag	c-Myc^{P1A} NLS	
147	ccgcctagtccagcttcacccgcttggcggcggc		
148	tattgatgaggcggcaaaagaacttccagatgctaatttgtag	wt Jα helix	
149	ccgcctacaaattagcatctggaagttcttttgccgcctcatc		
150	tttt**ggtctc**tgcccgggtgaagctggactagg	c-Myc^{P1AK4A} NLS	
151	tttt**ggtctc**tgggcggcggcggcaatattttctgc	c-Myc^{P1AK4A} NLS	
152	tttt**ggtctc**agaggccgccgccaagcgggtgaagctggac		*As*LOV2 I539E
153	tttt**ggtctc**acctcattttctgcagttttcttaatcagcatg		*As*LOV2 I539E
154	tttt**cgtctc**ctcgagtccgagcggagactctagag		
155	tttt**cgtctc**gaattcggtacccggtcacagcttgtctg		

in **Bold:** Restriction enzyme recognition sequences; underlined: overhangs after digest (only shown in case of *Bsa*I or *Bsm*B1 restriction sites)

Supplementary Methods

Plasmid construction

Plasmid construction was performed using standard restriction enzyme cloning. Phusion Flash High Fidelity PCR Master Mix (Thermo Scientific) was used for PCR amplifications. Oligonucleotides were ordered at Sigma Aldrich. A list of all vectors used and constructed in this study is shown in Supplementary Table S1. Oligonucleotide sequences used for PCR amplification or oligo cloning are shown in Supplementary Table S2.

mCherry-LINuS expression vectors based on monopartite NLSs

*As*LOV2 was derived from pTriEx-PA-Rac1[3] (kindly provided by Ilme Schlichting, MPI for Medical Research, Heidelberg). mCherry-LINuS variants bearing SV40-NLS derivatives were constructed by PCR amplification of *As*LOV2 truncations using forward primer 1 and one of the reverse primers 2-6 carrying the NLS sequence or wt Jα helix sequence as control. The resulting *As*LOV2-NLS fragments were digested *Bsr*GI/*Not*I.

For visualization of LINuS localization, mCherry was fused to the different LINuS variants using a standard 3x glycine-serine (GS) linker (GGSGGSGG). A yeast codon-optimized mCherry sequence[4] was amplified from pAA30 (kindly provided by Victor Sourjik, University of Heidelberg) using Primers 7 and 8 and the resulting fragment was digested *Eco*RI/*Bsr*GI. The mCherry fragment was then introduced alongside with the different *As*LOV2-NLS fragments into pSB1A3 (iGEM Registry of Standard Biological Parts) linearized with *Eco*RI/*Not*I. The resulting mCherry-*As*LOV2-NLS fragments were PCR-amplified with Primers 7 and 9, digested *Xba*I/*Xho*I and cloned between the *Xba*I and *Xho*I sites of the centromeric plasmid p413-TEF[5] for expression in yeast (yielding constructs pDB1-

5) and between *Nhe*I/*Xho*I sites of pcDNA3.1(-) (Invitrogen) for expression in mammalian cells (note that *Nhe*I and *Xba*I digestions yield compatible overhangs) (yielding construct pDB6-10).

To exchange the NLS in mammalian LINuS vectors, pDB6 was PCR-amplified with Primer 1 and one of the reverse primers 10-14 encoding NLSs c-Myc, c-Myc^{P1A}, E1A$_{pent}$, VP3 and SV40^{K4A} respectively. The resulting fragments were cloned into pDB6 with *Bsr*GI/*Xho*I, yielding constructs pDB11-15.

For the construction of LINuS variants with different degrees of Jα helix docking and stability[1], mutations were introduced into the *As*LOV2 domain by amplification with different sets of primers. The G528A mutation and the G528A/I532A double mutation were introduced in a first round of amplification using forward primer 1 and reverse primer 15 or 16 and the template pTriEx-PA-Rac1. The resulting amplicon was used as a template in a second round of amplification. Here, forward primer 17 was used to introduce the T406-7A mutation. Reverse primer 5 was used to introduce the SV40 NLS together with the N538A mutation and the reverse primers 11 and 18 were used to introduce the c-Myc^{P1A} NLS without or with the N538E mutation. The resulting fragments were ligated into pDB6 with *Bsr*GI/*Xho*I (yielding constructs pDB16-20).

NES sequences were introduced into pDB13 and pDB19 (mCherry-LINuS bearing the c-Myc^{P1A} NLS and either wt or T406-407A/G528A/N538E mutant *As*LOV2 domain) by PCR amplification with forward primers containing the sequence of the truncated NES from the heat stable protein kinase inhibitor[6] (PKIt; LALKLAGLDI; Primer 19) or the NES from the HIV-1 Rev protein[7] (HIV; LQLPPLERLTL; Primer 20) and reverse primers 11 (wt LOV2 domain) or 18 (mutated LOV2

domain). The resulting fragment was cloned into pcDNA3.1(-) with *Nhe*I/*Xho*I yielding constructs pDB21-23.

For C-terminal fusion of the PKIt NES, construct pDB13 was PCR-amplified with primers 7 and 21. The resulting fragment was cloned into pDB13 with *Xba*I/*Xho*I, thereby fusing the PKIt NES C-terminally to the c-Myc^{P1A} NLS, resulting in construct pDB24. Note that in the C-terminal fusion NLS and NES are separated by a 3x GS linker, while in the N-terminal NES fusion construct there is no linker between the NES and mCherry. An mCherry-LINuS construct carrying the c-Myc^{P1AK4A} NLS mutant impaired in importin binding was constructed by amplifying the complete vector pDB22 using primers 22 and 23 and re-ligating the PCR product after *Xho*I digest, yielding construct pJR25. In addition, an mCherry-LINuS construct carrying the wt *As*LOV2 Jα-helix was constructed by transferring a wt *As*LOV2 fragment from pDN85 (see below for details on construction) into pDB22 with *Bsr*GI/*Not*I, yielding construct pDN26.

N-terminal LINuS tagging

Primers 24 and 25 were used to amplify the *As*LOV2-c-Myc^{P1A} fragment from plasmid pDB22 and the resulting fragment was digested *Nhe*I/*Eco*RI. Primers 26 and 27 were used to amplify mCherry with an N–terminal 3x GS linker from the plasmid pAA30 and the resulting fragment was digested *Eco*RI/*Xho*I. The fragments were then combined in a three-way ligation with an NheI/XhoI linearized pcDNA3.1(-) vector fragment, yielding construct pDB27 encoding a *As*LOV2-c-Myc^{P1A} NLS-mCherry fusion.

CyclinB1-LINuS and CDK1-LINuS expression constructs

The pTreTight-CDK1AF-mCherry-LINuS plasmid was constructed by amplifying the PKIt-mCherry-*As*LOV2-c-Myc^{P1A} fragment from pDB22 using primers 28 and

29 followed by ligation into the pTreTight-CDK1AF plasmid (kindly provided by James E. Ferrell, Stanford University School of Medicine) using *Not*I/*Hind*III restriction sites, yielding construct pTD28. Note: CDK1AF corresponds to a constitutively active CDK1 version carrying the T14A/Y15F double mutation[8, 9]. We also created a construct for which the mCherry was exchanged for eYFP derived from pGal4-eYFP via unique *Not*I/*BsrG*I sites, resulting in construct pDN29.

The Cyclin B1-mCherry-LINuS fusion construct was constructed by PCR amplifying Cyclin B1 in two fragments from p1055-CyclinB1-FRB-YFP plasmid (kindly provided by James E. Ferrell, Stanford University School of Medicine) using primers 30/31 and 32/33 (note: primer 32 was used to insert the S147E mutation into Cyclin B1). Both fragments were digested *BsmB*I. Note: *BsmB*I cuts asymmetrically and enables creation of custom overhangs and thus scarless protein fusions. In this case a *Bam*HI-compatible overhang 5' of the first Cyclin B1 fragment and *Spe*I-compatible overhang 3' of the second fragment was created, as well as compatible overhangs for joining of the two fragments in a scareless manner. Fragments were then assembled in a three-way ligation into *Bam*HI/*Spe*I linearized LINuS-flex plasmid (pDN81, see below for details on construction) bearing c-Myc^{P1A} NLS and no NES, yielding construct pTD30. The Cyclin B1^{S147E}-mCherry-LINuS fusion was then cloned into pTreTight using *Nhe*I/*Mfe*I restriction sites, thereby creating pTreTight-CyclinB1 ^{S147E} -mCherry-LINuS (construct pDN31).

A bicistronic IRES vector for expression of CDK1AF-mCherry-LINuS and CyclinB1^{S147E}-mCherry-LINuS (construct pJR32) from the same promoter was created in two steps. We first incorporated an IRES sequence upstream of the CyclinB1^{S147E}-mCherry-LINuS coding sequence present in construct pTD30. To this aim, we PCR-amplified an IRES fragment from pCMV-YFP-IRES-neo (kindly

provided by Dirk Grimm, University of Heidelberg) using primers 34 and 35 and transferred it into pTD30 with *Bam*HI/*Sal*I. The so-obtained IRES-CyclinB1^{S147E}-mCherry-LINuS fragment was then transferred into pTD28 with *Sal*I/*Mfe*I, yielding the bicistronic construct pJR32.

mCherry-LINuS expression vectors based on bipartite NLSs

In order to replace the c-Myc^{P1A} NLS sequence present in mCherry-LINuS vector pDB22 carrying the PKIt NES with different bipartite NLS variants as well as bipartite NLS mutants, the complete pDB22 vector (excluding the c-Myc^{P1A} NLS) was PCR-amplified with primers 36 and 37 introducing *BsmB*I restriction sites. Following *BsmB*I digestion of the resulting PCR fragment, bipartite NLSs and bipartite NLS mutants were introduced as annealed oligo (oligo pairs 38-121), thereby generating mCherry-biLINuS1-30 (constructs pDN33-62) and mCherry-biLINuS2, 9 and 10 mutants m1-4 (constructs pDN63-74).

To exchange the NES in construct pDN34 (PKIt NES-mCherry-biLINuS2), NESs IkBα[10] (PSTRIQQQLGQLTLENLQ), MAPKK[10] (NLVDLQKKLEELELDEQQ), HIV-1[7] (LQLPPLERLTL), truncated HIV-1 (HIVt; LPPLERLTL) and a concatemer of two PKIt NES sequences separated by a GGSGGS linker (2xPKIt; LALKLAGLDIGGSGGSLALKLAGLDI) were introduced into the pDN34 vector. To this aim, the pDN34 construct (excluding the PKIt NES) was PCR-amplified with primers 122 and 123 containing *BsmB*I restriction sites. Following *BsmB*I digestion of the resulting PCR fragment, NESs were introduced as annealed oligos (oligo pairs 124-133), yielding constructs pDN75-79.

Constructs for LINuS-mediated reporter gene expression

LINuS-based transcription factor variants were cloned by first constructing a modular vector named LINuS-flex. It encodes a LINuS-regulated, synthetic fusion transcription factor composed of a LexA DNA binding domain[11] (derived from BBa_K165020, iGEM Registry of standard biological parts), an *E. coli* maltose binding protein[12] stuffer fragment (MBP; amplified from *E. coli* MG1655 genome), the VP64 transactivation domain[13] (derived from construct FKF1-VP16, kindly provided by Ricardo Dolmetsch, Stanford University) and the mCherry-*As*LOV2 (derived from mCherry-LINuS construct pDB22; the *As*LOV2 Jα helix was truncated after residue I539, thereby removing the c-Myc^{P1A} NLS). Notably, LINuS-flex was designed to contain a spacer sequence flanked by *Bsm*BI restriction sites between the VP64 transactivation domain and mCherry enabling scarless introduction of any NES sequence by standard oligo cloning. A second spacer sequence flanked by *Bsa*I restriction sites is located behind the truncated *As*LOV2 domain and enables scarless introduction of any NLS sequence by standard oligo cloning. A GS-rich linker separates the VP64 transactivation domain and mCherry. Unique *BamH*I and *Spe*I sites further enable the exchange of the LexA DBD-MBP-VP64 fragment by any sequence of interest. For assembly of LINuS-flex, the LexA DBD was PCR-amplified with primers 134 and 135 and digested *Bam*HI/*Bsa*I; MBP fragment 1 was PCR-amplified with primes 136 and 137 and digested *Bsa*I; MBP fragment 2 was PCR-amplified with primers 138 and 139 and digested *Bsa*I/*Kpn*I; mCherry-LINuS was PCR-amplified with primers 140 and 141 and digested *Not*I/*Xma*I; note: primers 140 and 141 introduce 2x*Bsa*I and 2x*BsmB*I sites for NLS and NES introduction into LINuS-flex on an oligo basis later on. A VP64 transactivation domain-encoding fragment was obtained by restriction digest of vector FKF1-VP16 with *Kpn*I/*Xma*I. The final LINuS-flex

construct (pDN80) was then assembled by performing a 6-way ligation into a *BamH*I/*Not*I linearized pmCherry-N1 backbone (Clontech).

Subsequently, NES PKIt and IkBα were introduced as annealed oligos (oligos 142-145) into LINuS-flex pre-digested with *Bsm*BI. Next, the NLS c-Myc^{P1A}, or the wild-type Jα helix sequence, were introduced into LINuS-flex without NES (pDN80) or its variants bearing PKIt and IkBα NESs described above as annealed oligos (oligos 146-149) via *Bsa*I, yielding constructs pDN81-86.

In order to introduce an additional K4A mutation into the c-Myc^{P1A} NLS present in constructs pDN81-83, the corresponding constructs were PCR-amplified with primers 150 and 151. Amplicons constituting the complete, linearized vectors now bearing the corresponding c-Myc^{P1AK4A} double mutant were digested with *Bsa*I and re-ligated in order to obtain constructs pDN87-89.

For construction of biLINuS transcription factor variants, the c-Myc^{P1A} NLS present in constructs pDN81-83 was exchanged by biNLS2, 9 or 10. To this aim, corresponding *As*LOV2-bipartite NLS-encoding fragments were obtained from constructs pDN34, pDN41 and pDN42 by *Not*I/*Bsr*GI restriction digestion and introduced into *Not*I/*Bsr*GI linearized constructs pDN81-83, thereby generating constructs pDN90-98.

In addition, a constitutively nuclear transcription factor positive control was cloned. Therefore, the complete construct pDN81 carrying the transcription factor variant with the c-Myc^{P1A} NLS and no NES was PCR-amplified with primers 152 and 153. These primers introduce the I539E mutation locking the *As*LOV2 domain in a pseudolit state[2]. The PCR product was digested with *Bsa*I and re-ligated, yielding construct pDN99 that is active independent of any light induction.

A firefly luciferase reporter driven from a minimal promoter with 4 tandem repeats of LexA DNA binding sites was obtained by replacing the 5x Gal4 binding sites in pFR-Luc (Agilent) with a 4x LexA DNA binding site containing fragment (derived from BBa_K165031, iGEM Registry of standard biological parts). Therefore, a fragment comprising the complete pFR-Luc vector but lacking the Gal4 binding sites was PCR-amplified with primers 154 and 155. The primers introduced *BsmB*I restriction sites yielding *Eco*RI/*Xho*I compatible overhangs after restriction digest. A 4x LexA DNA binding site fragment was obtained by restriction digestion of vector BBa_K165031 (iGEM Registry of standard biological parts) with *Eco*RI/*Xho*I and the resulting fragment was ligated with the pre-digested pFR-Luc backbone fragment, thereby generating construct pDN100.

Supplementary References

1. Strickland, D. et al. TULIPs: tunable, light-controlled interacting protein tags for cell biology. *Nature methods* **9**, 379-384 (2012).
2. Harper, S.M., Christie, J.M. & Gardner, K.H. Disruption of the LOV-Jalpha helix interaction activates phototropin kinase activity. *Biochemistry* **43**, 16184-16192 (2004).
3. Wu, Y.I. et al. A genetically encoded photoactivatable Rac controls the motility of living cells. *Nature* **461**, 104-108 (2009).
4. Khmelinskii, A., Meurer, M., Duishoev, N., Delhomme, N. & Knop, M. Seamless gene tagging by endonuclease-driven homologous recombination. *PloS one* **6**, e23794 (2011).
5. Mumberg, D., Muller, R. & Funk, M. Yeast vectors for the controlled expression of heterologous proteins in different genetic backgrounds. *Gene* **156**, 119-122 (1995).
6. Wen, W., Taylor, S.S. & Meinkoth, J.L. The expression and intracellular distribution of the heat-stable protein kinase inhibitor is cell cycle regulated. *The Journal of biological chemistry* **270**, 2041-2046 (1995).
7. Fischer, U., Huber, J., Boelens, W.C., Mattaj, I.W. & Luhrmann, R. The HIV-1 Rev activation domain is a nuclear export signal that accesses an export pathway used by specific cellular RNAs. *Cell* **82**, 475-483 (1995).
8. Ma, Y., Yuan, X., Wyatt, W.R. & Pomerening, J.R. Expression of constitutively active CDK1 stabilizes APC-Cdh1 substrates and potentiates premature spindle assembly and checkpoint function in G1 cells. *PloS one* **7**, e33835 (2012).
9. Pomerening, J.R., Ubersax, J.A. & Ferrell, J.E., Jr. Rapid cycling and precocious termination of G1 phase in cells expressing CDK1AF. *Molecular biology of the cell* **19**, 3426-3441 (2008).
10. Henderson, B.R. & Eleftheriou, A. A comparison of the activity, sequence specificity, and CRM1-dependence of different nuclear export signals. *Experimental cell research* **256**, 213-224 (2000).
11. Hurstel, S., Granger-Schnarr, M., Daune, M. & Schnarr, M. In vitro binding of LexA repressor to DNA: evidence for the involvement of the amino-terminal domain. *The EMBO journal* **5**, 793-798 (1986).
12. Marshall, K.S., Zhang, Z., Curran, J., Derbyshire, S. & Mymryk, J.S. An improved genetic system for detection and analysis of protein nuclear import signals. *BMC molecular biology* **8**, 6 (2007).
13. Yazawa, M., Sadaghiani, A.M., Hsueh, B. & Dolmetsch, R.E. Induction of protein-protein interactions in live cells using light. *Nature biotechnology* **27**, 941-945 (2009).
